# Design and Synthesis of New 6-Nitro and 6-Amino-3,3a,4,5-Tetrahydro-2*H*-Benzo[*g*]indazole Derivatives: Antiproliferative and Antibacterial Activity

**DOI:** 10.3390/molecules24234236

**Published:** 2019-11-21

**Authors:** Viviana Cuartas, María del Pilar Crespo, Eva-María Priego, Leentje Persoons, Dirk Daelemans, María-José Camarasa, Braulio Insuasty, María-Jesús Pérez-Pérez

**Affiliations:** 1 Grupo de Investigación de Compuestos Heterocíclicos, Departamento de Química, Universidad del Valle, A. A. Cali 25360, Colombia; viviana.cuartas@correounivalle.edu.co; 2 Centre for Bioinformatics and Photonics-CIBioFI, Calle 13 No. 100-00, Edificio E20, No. 1069, Cali 760032, Colombia; 3 Grupo de Biotecnología e Infecciones Bacterianas, Departamento de Microbiología, Universidad del Valle, Cali 760043, Colombia; maria.crespo.ortiz@correounivalle.edu.co; 4 Instituto de Química Médica (IQM, CSIC), Juan de la Cierva 3, 28006 Madrid, Spain; empriego@iqm.csic.es (E.-M.P.); mj.camarasa@iqm.csic.es (M.-J.C.); 5 KU Leuven Department of Microbiology, Immunology and Transplantation, Laboratory of Virology and Chemotherapy, Rega Institute for Medical Research, KU Leuven, Herestraat 49, 3000 Leuven, Belgium; leentje.persoons@kuleuven.be (L.P.); dirk.daelemans@kuleuven.be (D.D.)

**Keywords:** indazole, 2-benzylidene-1-tetralone, antiproliferative activity, antibacterial activity

## Abstract

New substituted benzo[g]indazoles functionalized with a 6-nitro and 6-amino groups have been synthesized by the reaction of benzylidene tetralones with hydrazine in acetic acid. The resulting conformationally-constrained compounds were evaluated for their antiproliferative activity against selected cancer cell lines. The nitro-based indazoles **11a**, **11b**, **12a** and **12b** have shown IC_50_ values between 5–15 μM against the lung carcinoma cell line NCI-H460. Moreover, the nitro compounds were tested for antibacterial activity where compounds **12a** and **13b** have shown MIC values of 250 and 62.5 μg/mL against *N. gonorrhoeae* with no hemolytic activity in human red blood cells (RBC).

## 1. Introduction

Indazoles are benzo-fused pyrazoles for which a broad range of biological properties have been described [1,2]. Fused tricyclic pyrazole derivatives have been studied as necroptosis inhibitors [3,4], high affinity ligands for the human dopamine D4 receptor [5], phosphodiesterase 4 (PDE4) inhibitors [6], cannabinoid-2 receptor ligands [7], inhibitors of β-hematin formation conferring *in vitro* antimalarial activity [8] or inhibition of isocitrate dehydrogenase 1 (IDH1) [9]. Moreover, the tricyclic pyrazole core is present in compounds with antimicrobial [10,11,12] and antiproliferative activity [13,14,15]. The most widely used synthetic approach towards these compounds involves the reaction of α,β-unsaturated ketones and hydrazines [6,8,10,11,12,14]. Very recently, a Lewis acid promoted reaction of cycloalkanones with hydrazones has also been described to synthesize 2,3-diaryl-4,5,6,7-tetrahydro-1*H*-indazoles [16]. It should be highlighted that these fused tricyclic pyrazole derivatives are characterized by their conformational restriction [15].

**Figure 1:**
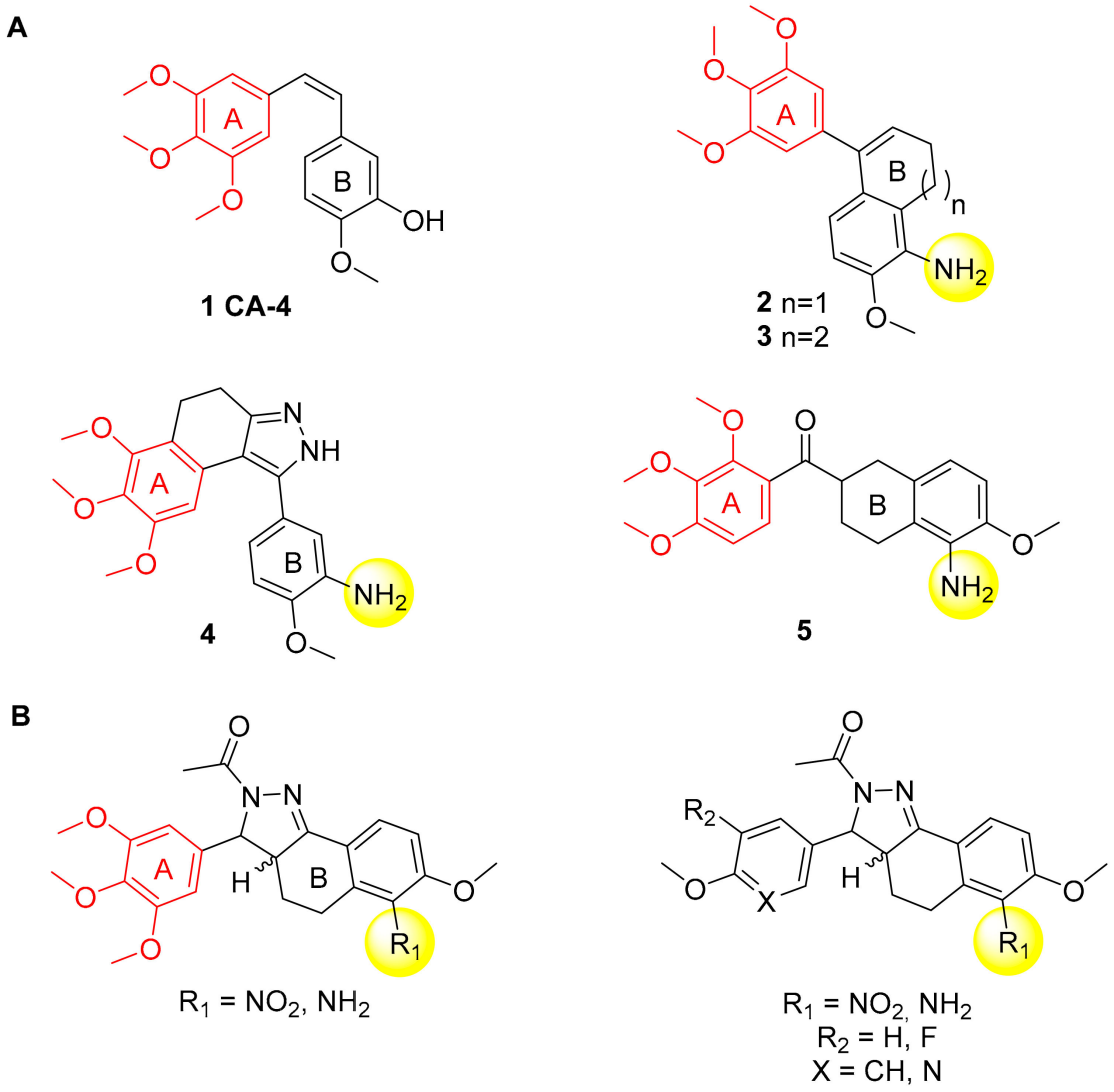
(**A**) Structure of **CA-4** and recently reported conformationally restricted colchicine binding site inhibitors; (**B**) tricyclic pyrazole derivatives here addressed.

Colchicine-site inhibitors based on combretastatin A-4 (**1**, **CA-4**, Figure 1A) have been extensively studied as antimitotic and vascular disrupting agents [17,18,19]. Among the different approaches followed to mimic the *cis* (active) configuration of **CA-4**, the design and synthesis of conformationally restricted ligands have led to very potent inhibitors. Among recently reported examples, the dihydronaphthalene and benzosuberene derivatives (**2** and **3**, respectively, Figure 1A) have shown antiproliferative activity at the sub nM range [20]. Very recently, Jiang, J. et al. [21] described a novel 1-phenyl-dihydrobenzoindazole (**4**, Figure 1A) with a locked conformation, that inhibited tubulin polymerization with an IC_50_ of 1.6 μM and showed antitumor properties against a human colon cancer cell line with an IC_50_ value of 1 nM. In both series the presence of an amino group on ring B is meant to mimic the phenolic OH of **CA-4** and this amino group has been used for the synthesis of prodrugs thereof [20,21]. In addition, in all cases a trimethoxyphenyl is present as ring A (Figure 1).

We have recently reported on tetrahydronaphtalene derivatives as conformational restricted mimetics of chalcone derivatives that also bind the colchicine binding site in tubulin [22]. As in the previous examples, an amino group was incorporated at position 5 on ring B and this was shown to be crucial for the antiproliferative activity and the tubulin binding capacity. Thus, compound **5** was found to inhibit CEM (human lymphoblastic leukemia) proliferation at the sub µM range.

Based on the general interest of tetrahydroindazole derivatives, we have here designed and synthesized a new series of tricyclic pyrazoline derivatives incorporating an amino group at position 6 on ring B that were obtained through reduction of their nitro precursors (Figure 1B). Several 4-methoxy aryl rings have been incorporated as ring A. In particular, those compounds with a 3,4,5-trimethoxyphenyl as ring A and an amino group at position 6 on ring B could mimic the conformation of our colchicine-binding site inhibitor 5, as will be later discussed. The synthesized compounds have been tested for their antiproliferative activity against a wide panel of tumor cells. As a part of our continuing effort to find molecules with antibacterial activity [23,24,25,26], the nitro derivatives were also screened against wild type and multidrug-resistant bacteria, particularly those frequently found in relevant human infections.

## 2. Results and Discussion

### 2.1. Chemistry

**Scheme 1:**
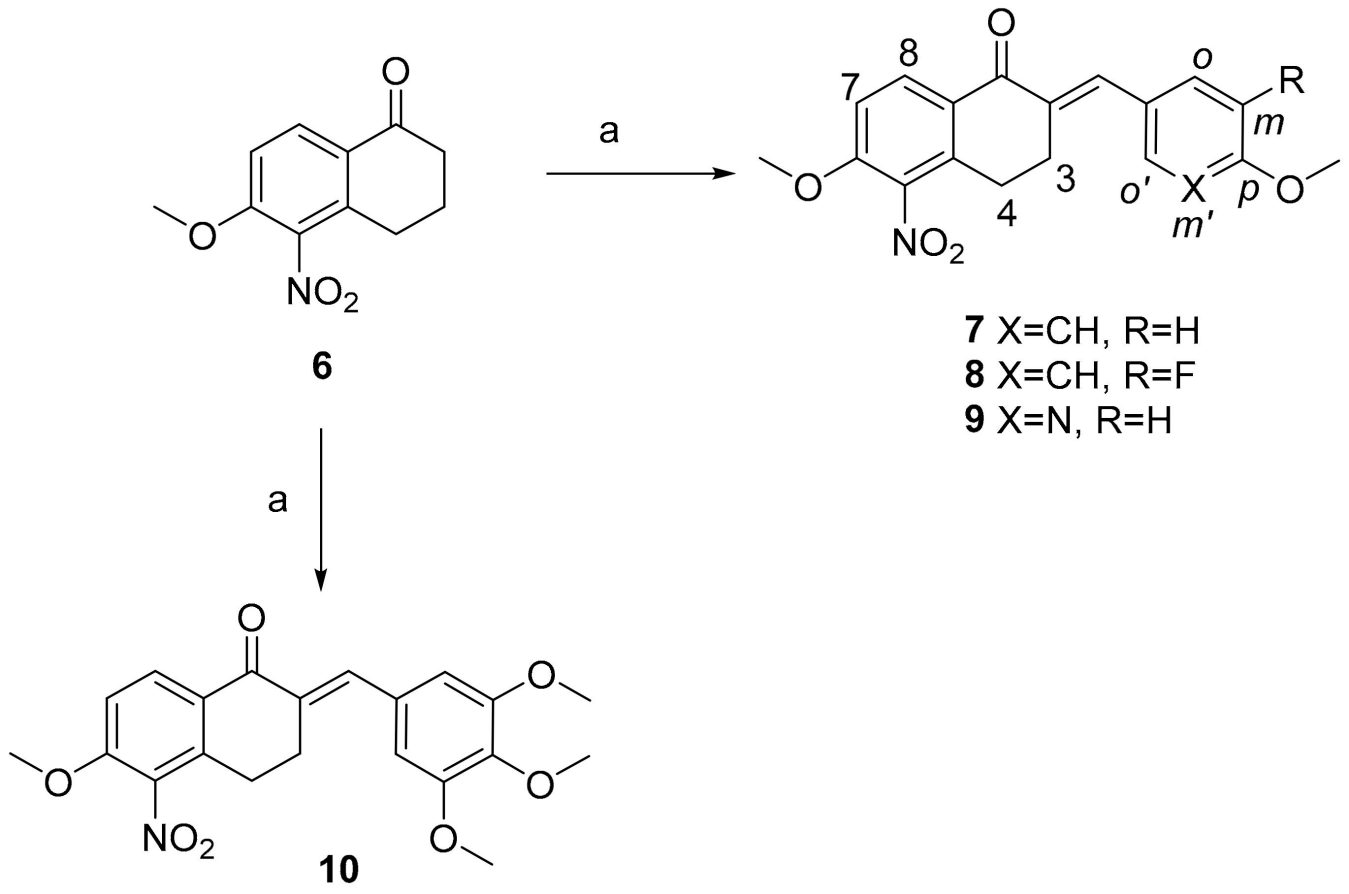
Synthesis of the substituted chalcones **7**–**10**. Reagents and conditions: (a) aromatic aldehyde, NaOH, EtOH, rt., 7 h.

The synthesis of the targeted compounds showed in Figure 1B required the 6-methoxy-5-nitro-1-tetralone (**6**) as a starting material that was prepared as previously described [20,27]. Reaction of **6** by Claisen-Schmidt condensation with aromatic aldehydes (Scheme 1) in the presence of NaOH in ethanol provided the corresponding chalcones **7**–**10** in excellent yields (82, 86, 82 and 77% yield, respectively).

**Scheme 2:**
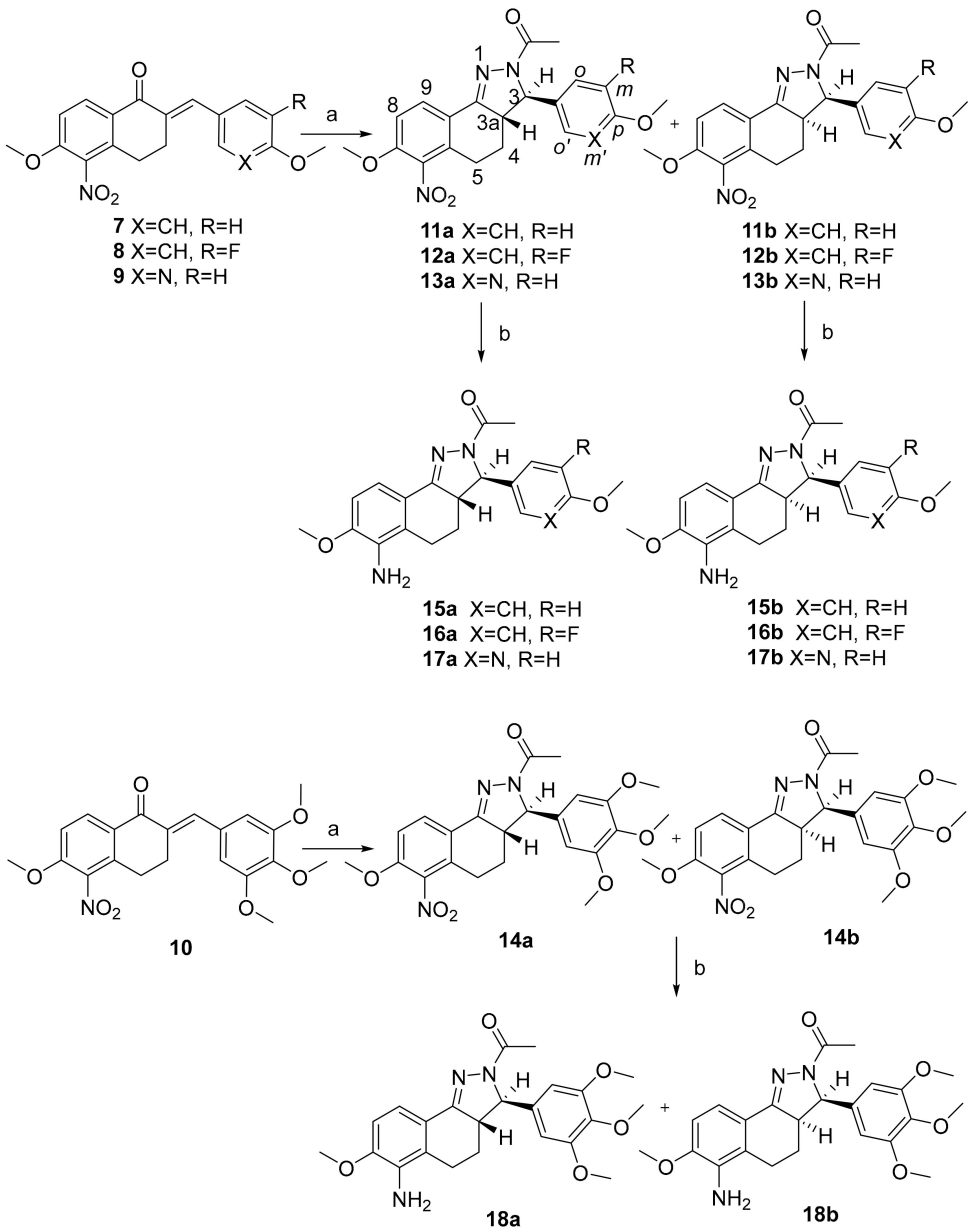
General synthesis of tricyclic derivatives **11**–**18a,b**. Reagents and conditions: (a) NH_2_NH_2_·H_2_O, AcOH, reflux, 2 h. (b) Fe powder, HCl, EtOH:H_2_O, reflux, 1 h.

The chalcones **7**–**9** were then treated with hydrazine hydrate in the presence of acetic acid to give a mixture of diastereoisomers **11a**–**13a** and **11b**–**13b** in a 1:1 ratio that could be separated by chromatography, and with global yields of 59, 74 and 71%, respectively (Scheme 2). The structural and stereochemical assignment of each pair of enantiomers was performed based on NMR experiments. As an example, the 2D NOESY spectrum for compound **11b** showed a NOE signal between the protons H-3 and H-3a, and therefore **11b** was assigned as the (3*R*,3a*R*)-*rel* stereoisomer. In addition, the signal corresponding to H-3 in **11b** has a coupling constant (*J_3,3a_* = 11.0 Hz) larger than the one of the same signal in the diastereomer **11a** (*J_3,3a_* = 9.7 Hz). When the ^13^C NMR spectra of **11a** and **11b** were compared, the signal corresponding to C-3a appeared at 53.8 and 46.9 ppm, respectively. Thus, there is a significant upfield shift for this signal in **11b**, suggesting the *cis* configuration ((3*R*,3a*R*)-*rel*), in agreement with those reported in the literature [8,10,12,28]. Interestingly, in the whole series, one of the signals corresponding to H-4 for the (3*R*,3a*R*)-*rel* isomers showed a chemical shift around 0.7–0.8 ppm, probably due to the shielding effect of the aromatic ring A, while this signal for the (3*R*,3a*S*)-*rel* isomers is around 1.90 ppm. We consider that this difference can also be employed as useful and convenient criteria to assign both isomers. Similarly, reaction of chalcone **10** with hydrazine hydrate in the presence of acetic acid gave a mixture of the two trimethoxy derivatives **14a** and **14b** (Scheme 2), in a global yield of 66%, but whose separation by chromatography was very laborious. Thus, the mixture of **14a** and **14b** was used for the next reaction.

Finally, the nitro derivatives **11**–**14a,b** were reduced to their amino analogs by treatment with Fe in an EtOH:H_2_O mixture at reflux in the presence of HCl (Scheme 2), following the procedure previously used for the reduction of the 5-nitrotetrahydronaphthalenes [22], providing compounds **15a**–**18a** and **15b**–**18b** in moderate to good yields (25–75%). These compounds **15a**–**18a** and **15a**–**18b** showed similar spectroscopic data and therefore, we discuss here compound **15a** as the representative of this series. In the ^1^H-NMR spectrum, the signal corresponding to H-3a appears as a double-double-doublet at 3.05 ppm with coupling constants ^3^*J* = 13.8, 9.2, 4.8 Hz; the signals for H-4 appear as two multiplets at 1.83 ppm integrating for one proton and between 2.15 and 2.17 ppm for four protons corresponding to H-4 and the methyl protons of acetyl group. A broad singlet for amino protons was observed at 4.61 ppm. The ^13^C-NMR spectrum showed the expected nineteen signals for the carbons of compound **15a**.

**Figure 2:**
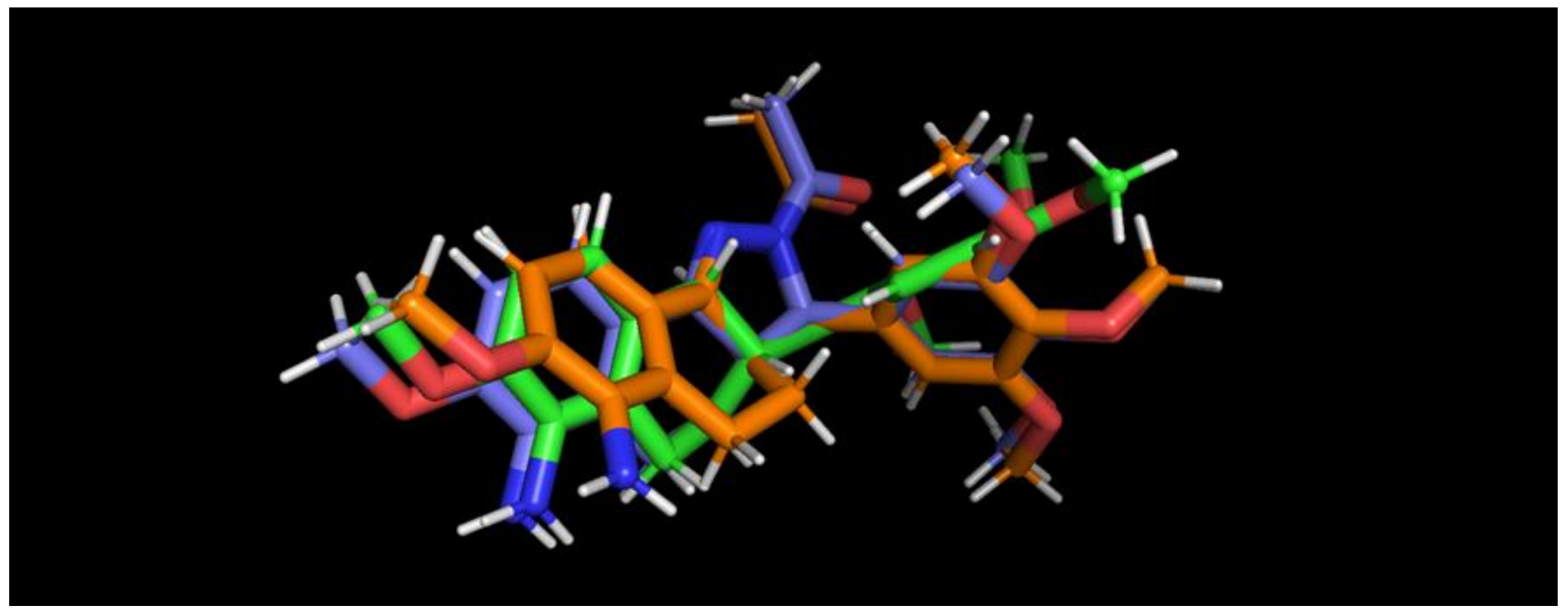
Overlap of the 3D structures of isomers **18a** (3*R*,3a*S*) (purple), **18b** (3*R*,3a*R*) (orange) and compound **5** (*R*) (green).

As mentioned in the introduction, these compounds are characterized by a high degree of conformational restriction, thus they could represent an alternative scaffold for colchicine site binding agents. Thus, 3D structures of compounds **18a** and **18b** were constructed and their geometries were optimized with MOPAC2012 (AM1 method) [29]. This was followed by a manual superposition of the geometry-optimized structures with the conformation of the tetrahydronaphtalene derivative **5** for which we have recently reported its tubulin binding capacity and antiproliferative activity [22]. As shown in Figure 2, the degree of overlapping is quite relevant, the major differences being the relative orientation of ring A and the presence of the additional acetylated pyrazoline ring.

**Figure 3:**
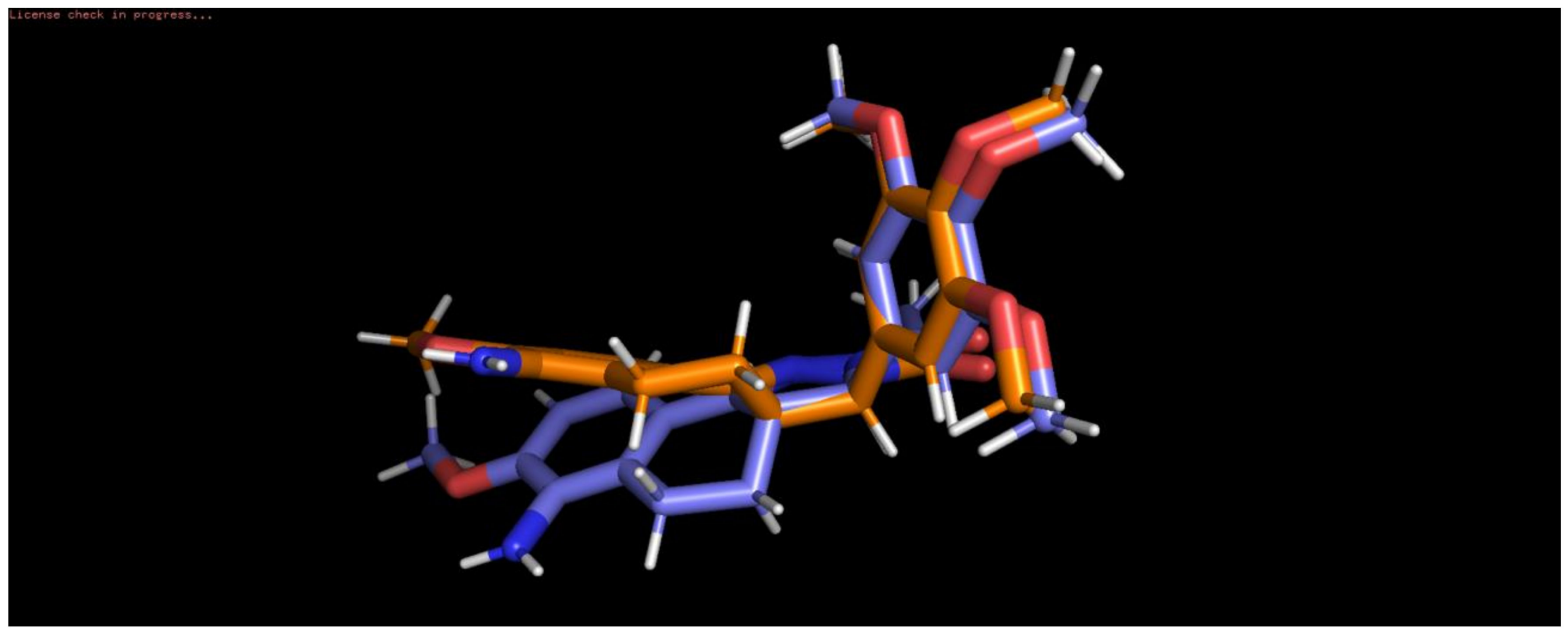
Compound **18a** with a (3*R*,3a*S*) configuration is shown in purple while compound **18b** with a (3*R*,3a*R*) configuration is shown in orange.

Interestingly, a comparison of the 3D structures of **18a** (3*R*,3a*S*) and **18b** (3*R*,3a*R*) also helps to explain the differences observed in the chemical shifts in the ^1^H-NMR spectrum of one of the protons H-4. As shown in Figure 3, for **18b** one of the H-4 protons is facing the phenyl ring A. Thus, this shielding effect explains the differences in chemical shifts for this proton between the **b**-isomers (0.7–0.8 ppm) and the **a**-isomers (around 1.90 ppm) in the whole series.

### 2.2. Biological Evaluation

#### 2.2.1. Antiproliferative Activity

All benzo[*g*]indazole derivatives **11**–**18a,b** were tested for their antiproliferative activity against a wide panel of tumor cells. As shown in Table 1, most of the 3,3a,4,5-tetrahydro-2*H*-benzo[*g*]indazole derivatives showed modest antiproliferative activity against the cell lines tested. In particular, the trimethoxy derivatives **18a** and **18b** showed IC_50_ values around 50 µM. On the other hand, the 6-nitro derivatives **11a**, **11b** and **12a**, **12b**, showed IC_50_ values in the low µM range against a lung carcinoma cell line NCI-H460. Some additional studies were performed for compounds **11a**, **11b**, and **12a**, **12b**. At 10 µM, the compounds did not affect tubulin as measured by immunostaining in HeLa cells, suggesting that tubulin is not the target for the antiproliferative activity. They were also screened for DNA intercalation or DNA damage, using standardized procedures. These protocols are included as Supplementary Materials. Also, in this case, no significant effect was observed.

#### 2.2.2. Antibacterial Activity

Compounds **7**–**10** and **11**–**14a,b** were investigated for their antibacterial activity against wild type and multidrug-resistant bacteria including methicillin-susceptible *S. aureus* ATCC 25923 (MSSA), methicillin-resistant *S. aureus* ATCC 43300 (MRSA), vancomycin-intermediate *S. aureus* (VISA), *E. coli* ATCC 25922, *P. aeruginosa* ATCC 27853, *K. pneumoniae* ATCC 700603 (Extended spectrum beta-lactamase positive, ESBL+), *K. pneumoniae* BAA1705 (carbapenemase-positive) and *N. gonorrhoeae* ATCC 49226. The minimum inhibitory concentration (MIC) was determined by broth microdilution in those compounds with reproducible antimicrobial effect.

Table 2 shows that *N. gonorrhoeae* growth was inhibited by **12a** and **13b**. Compound **13b** was the most active of the series with a MIC value of 62.5 μg/mL, whereas **12a** induced moderate growth inhibition (MIC = 250 μg/mL). Comparison of the antibacterial activity of the isomers **13a** (3*R*,3a*S*)-*rel*- (MIC ≥ 500 μg/mL) and **13b** (3*R*,3a*R*)-*rel*- (MIC = 62.5 μg/mL) against *N. gonorrhoeae* shows that the configuration of the molecule has an important effect on the activity. On the other hand, it is worth to take into account the recent emergence of resistant clinical isolates of *N. gonorrhoeae* to a variety of antimicrobial agents, for which the need to find new antigonococcal agents is of paramount importance [30,31,32]. Compounds **12a** and **13b** were further tested for hemolytic activity or toxicity against RBC. None of the active compounds induced hemolysis of the human RBC.

## 3. Materials and Methods

### 3.1. General Information

Reagents and solvents used were purchased from Sigma-Aldrich (St. Louis, MO, USA). Reactions were monitored by TLC on silica gel 60 F_254_ aluminum plates (Merck, Dramstand, Germany). Spots were detected under UV light (254 nm) and/or charring with ninhydrin or phosphomolybdic acid. Melting points were measured on a M170 apparatus (Mettler Toledo, Columbus, Ohio, USA) and are uncorrected. The elemental analyses were performed using a CHN-O-RAPID instrument (Heraeus, Hanau, Germany). Analyses indicated by the symbols of the elements or functions were within ±0.4% of the theoretical values. ^1^H and ^13^C-NMR spectra were run on an INOVA-300 instrument (Varian, now Agilent, Santa Clara, CA, USA) operating at 300 (^1^H) and 75 MHz (^13^C) and a Varian INOVA-400 operating at 400 (^1^H) and 100 MHz (^13^C), respectively. Chemical shift (δ) values are reported in parts per million (ppm). Separations on silica gel were performed by preparative centrifugal circular thin-layer chromatography (CCTLC) on a Chromatotron^R^ (Kiesegel 60 PF_254_ gipshaltig, Merck), with a layer thickness of 1 and 2 mm and flow rate of 4 or 8 mL/min, respectively. Flash chromatography was performed on silica gel as stationary phase. HPLC analysis was performed on an Agilent 1120 compact LC system (Santa Clara, CA, USA), equipped with an ACE 5 C18-300 column (15 cm × 4.6 mm), UV detection, and flow rate 1 mL/min, using as mobile phase A H_2_O (containing 0.1% TFA) and as mobile phase B acetonitrile. Retention times (T_R_) are reported in minutes. For all compounds, HPLC purity was determined to be greater than 95%. Compounds were also analyzed by HPLC/MS with an e2695 LC (Waters, Milford, Massachusetts, USA) coupled to a Waters 2996 Photodiode Array Detector and a Waters Micromass ZQ. The column used is a Waters SunFire C18 2.1 × 50 mm, 3.5 µm, and the mobile phases were A: acetonitrile and B: H_2_O, together with a constant 5% of C (H_2_O with 2% formic acid) to assure a 0.1% of formic acid along the run.

### 3.2. Chemistry

#### 3.2.1. Preparation of 6-Methoxy-5-nitro-1-tetralone (**6**)

To a solution of 6-methoxy-1-tetralone (600 mg, 3.40 mmol) in acetic anhydride (3.0 mL) in an ice bath, a mixture of 65% HNO_3_ (330 µL) in acetic acid (210 µL) was added dropwise over 2 h, and then the mixture was stirred for additional 2 h at 0 °C. The resulting solid was treated with H_2_O (20 mL) and extracted with EtOAc (3 × 30 mL). The combined organic layers were dried over anhydrous Na_2_SO_4_, filtered and evaporated under reduced pressure. The residue was purified by silica gel chromatography (hexane:EtOAc 8:2) to provide 252 mg (33%) of 6-methoxy-5-nitro-1-tetralone, and 233 mg (31%) of 6-methoxy-7-nitro-1-tetralone as light yellow solids, respectively. Data for 6-methoxy-5-nitro-1-tetralone: ^1^H-NMR (DMSO-*d*_6_, 400 MHz) δ: 2.05 (p, *J* = 6.5 Hz, 2H), 2.60 (t, *J* = 6.5 Hz, 2H), 2.78 (t, *J* = 6.5 Hz, 2H), 3.97 (s, 3H, OCH_3_), 7.36 (d, *J* = 8.9 Hz, 1H, Ar), 8.08 (d, *J* = 8.9 Hz, 1H, Ar). Mp 166–167 °C. ^1^H-NMR data are similar to those previously described [20,27].

#### 3.2.2. General Procedure for the Synthesis of Chalcones **7**–**10** Derived from 6-Methoxy-5-Nitro-1-Tetralone (General Procedure A)

A mixture of 6-methoxy-5-nitro-1-tetralone (**6**) (1.0 eq), the selected aldehyde (1.2 eq) and a pellet of NaOH (185 mg) in ethanol was stirred at room temperature for 7 h. The solid thus formed was filtered and washed with ethanol and water to provide the chalcones **7** and **8** as pure yellow solids. For **9** and **10** purifications by flash column chromatography were required and performed as specified.

(*E*)-6-Methoxy-2-(4-methoxybenzylidene)-5-nitro-3,4-dihydronaphthalen-1(*2H*)-one (**7**), following the general procedure A, 6-methoxy-5-nitro-1-tetralone (**6**) (100 mg, 0.45 mmol) reacted with *p*-anisaldehyde (79 mg, 0.58 mmol) in ethanol (3.0 mL). The precipitate was collected by filtration and washed with ethanol and water to give 125 mg (81% yield) of **7**, which did not require further purification. Mp 171–173 °C. MS (ES, positive mode): *m*/*z* 340 [M + H]^+^. ^1^H-NMR (DMSO-*d_6_*, 400 MHz) δ: 2.79 (t, *J* = 6.5 Hz, 2H, H-4), 3.11 (t, *J* = 6.5 Hz, 2H, H-3), 3.81 (s, 3H, OCH_3_), 3.99 (s, 3H, OCH_3_), 7.03 (d, *J* = 8.7 Hz, 2H, H*_m_*), 7.40 (d, *J* = 8.9 Hz, 1H, H-7), 7.51 (d, *J* = 8.7 Hz, 2H, H_o_), 7.70 (s, 1H, H_β_), 8.17 (d, *J* = 8.9 Hz, 1H, H-8). ^13^C-NMR (DMSO-*d_6_*, 100 MHz) δ: 22.8 (C-4), 25.6 (C-3), 55.3 (OCH_3_), 57.1 (OCH_3_), 112.1 (C-7), 114.2, 126.3, 127.4, 131.6, 131.7 (C-8), 132.0, 135.6, 136.5 (C_β_), 139.0, 153.7, 159.9, 184.1 (C=O). Analytical HPLC (gradient 30–95% acetonitrile in 10 min): T_R_: 8.44; area: 98%. Anal. cal. for (C_19_H_17_NO_5_.0.5H_2_O): C, 65.51; H, 5.21, N, 4.02. Found: C, 65.94; H, 4.93, N, 4.23.

(*E*)-2-(3-Fluoro-4-methoxybenzylidene)-6-methoxy-5-nitro-3,4-dihydronaphthalen-1(2*H*)-one (**8**), following the general procedure A, 6-methoxy-5-nitro-1-tetralone (**6**) (150 mg, 0.68 mmol) reacted with 3-fluoro-4-methoxybenzaldehyde (128 mg, 0.83 mmol) in ethanol (3.0 mL). The precipitate was collected by filtration and washed with ethanol and water to give 208 mg (86% yield) of **8** as a yellow solid, which did not require further purification. Mp 189–190 °C. MS (ES, positive mode): *m*/*z* 358 [M + H]^+^. ^1^H-NMR (DMSO-*d_6_*, 400 MHz) δ: 2.80 (t, *J* = 6.5 Hz, 2H, H-4), 3.10 (t, *J* = 6.5 Hz, 2H, H-3), 3.89 (s, 3H, OCH_3_), 3.99 (s, 3H, OCH_3_), 7.25 (t, *J* = 8.8 Hz, 1H, H_m’_), 7.36 (dd, *J* = 8.4, 2.4 Hz, 1H, H_o’_), 7.40 (d, *J* = 8.9 Hz, 1H, H-7), 7.44 (dd, *J* = 12.8, 2.1 Hz, 1H, H_o_), 7.66 (s, 1H, H_β_), 8.17 (d, *J* = 8.9 Hz, 1H, H-8). ^13^C-NMR (DMSO-*d_6_*, 100 MHz) δ: 22.7 (C-4), 25.5 (C-3), 56.1 (OCH_3_), 57.2 (OCH_3_), 112.1 (C-7), 113.8 (d, ^3^*J_C-F_* = 2.2 Hz, C_m’_), 117.3 (d, ^2^*J_C-F_* = 18.5 Hz, C_o_), 126.1, 127.3 (d, ^4^*J_C-F_* = 3.2 Hz, C*_o’_*), 127.8 (d, ^3^*J_C-F_* = 6.9 Hz, C), 131.7 (C-8), 132.9, 135.2 (d, ^4^*J_C-F_* = 2.1 Hz, C_β_), 135.6, 139.0, 147.8 (d, ^2^*J_C-F_* = 10.6 Hz, C-OCH_3_), 151.1 (d, ^1^*J_C-F_* = 244.3 Hz, C-F), 153.7, 184.0 (C=O). Analytical HPLC (gradient 30–95% acetonitrile in 10 min): T_R_: 8.33; area: 97%. %. Anal. cal. for (C_19_H_16_FNO_5_): C, 63.86; H, 4.51, N, 3.92. Found: C, 63.72; H, 4.55, N, 3.78.

(*E*)-6-Methoxy-2-((6-methoxypyridin-3-yl)methylene)-5-Nitro-3,4-Dihydronaphthalen-1(2*H*)-one (**9**), following the general procedure A, 6-methoxy-5-nitro-1-tetralone (**6**) (100 mg, 0.45 mmol) reacted with 6-methoxy-3-pyridinecarboxaldehyde (76 mg, 0.55 mmol) in ethanol (3.0 mL). The precipitate was collected by filtration and washed with ethanol and water. The solid obtained was purified by flash column chromatography (CH_2_Cl_2_: MeOH 10:0.1) to give 125 mg (82% yield) of **9** as a yellow solid. Mp 176–178 °C. MS (ES, positive mode): *m*/*z* 341 [M + H]^+^. ^1^H-NMR (DMSO-*d_6_*, 400 MHz) δ: 2.81 (t, *J* = 6.5 Hz, 2H, H-4), 3.09 (t, *J* = 6.5 Hz, 2H, H-3), 3.90 (s, 3H, OCH_3_), 3.99 (s, 3H, OCH_3_), 6.92 (d, *J* = 8.7 Hz, 1H, H_m_), 7.41 (d, *J* = 8.9 Hz, 1H, H-7), 7.69 (s, 1H, H_β_), 7.91 (dd, *J* = 8.7, 2.5 Hz, 1H, H_o_), 8.18 (d, *J* = 8.9 Hz, 1H, H-8), 8.39 (d, *J* = 2.4 Hz, 1H, H_o’_). ^13^C-NMR (DMSO-*d_6_*, 75 MHz) δ: 23.2 (C-4), 26.0 (C-3), 53.8 (OCH_3_), 57.5 (OCH_3_), 110.9, 112.5 (C-7), 124.9, 126.5, 132.1 (C-8), 133.4, 133.8, 136.1 (C_β_), 139.4, 140.6, 149.5, 154.2, 163.8, 184.2 (C=O). Analytical HPLC (gradient 30–95% acetonitrile in 10 min): T_R_: 7.61; area: 95%. %. Anal. cal. for (C_18_H_16_N_2_O_5_): C, 63.53; H, 4.74; N, 8.23. Found: C, 63.42; H, 4.89, N, 8.11.

(*E*)-6-Methoxy-5-Nitro-2-(3,4,5-trimethoxybenzylidene)-3,4-Dihydronaphthalen-1(2*H*)-one (**10**), following the general procedure A, 6-methoxy-5-nitro-1-tetralone (**6**) (250 mg, 1.13 mmol) reacted with 3,4,5-trimethoxybenzaldehyde (276 mg, 1.41 mmol) in ethanol (5.0 mL). The precipitate was collected by filtration and washed with ethanol and water. The solid obtained was purified by flash column chromatography (CH_2_Cl_2_) to give 347 mg (77% yield) of **10** as a yellow solid. Mp 204–205 °C. MS (ES, positive mode): *m*/*z* 400 [M + H]^+^. ^1^H-NMR (DMSO-*d_6_*, 400 MHz) δ: 2.81 (t, *J* = 6.5 Hz, 2H, H-4), 3.17 (t, *J* = 6.5 Hz, 2H, H-3), 3.71 (s, 3H, OCH_3_), 3.81 (s, 6H, OCH_3_), 3.99 (s, 3H, OCH_3_), 6.84 (s, 2H, H_o_), 7.41 (d, *J* = 9.0 Hz, 1H, H-7), 7.70 (s, 1H, H_β_), 8.18 (d, *J* = 9.0 Hz, 1H, H-8). ^13^C-NMR (DMSO-*d_6_*, 75 MHz) δ: 22.9 (C-4), 25.7 (C-3), 56.0 (OCH_3_), 57.2 (OCH_3_), 60.1 (OCH_3_), 107.7 (C_o_), 112.2 (C-7), 126.2, 130.4, 131.8 (C-8), 133.2, 135.7, 136.8 (C_β_), 138.3, 139.1, 152.8, 153.8, 184.1 (C=O). Analytical HPLC (gradient 30–95% acetonitrile in 10 min): T_R_: 8.00; area: 98%. Anal. cal. for (C_21_H_21_NO_7_): C, 63.15; H, 5.30, N, 3.51. Found: C, 63.08; H, 5.30, N, 3.64.

#### 3.2.3. General Procedure for the Synthesis of *N*-Acetylpyrazolines **11**–**14a,b** (General Procedure B)

To a solution of the selected chalcone (**7**–**10**) (1.0 eq) in acetic acid (2.0 mL), hydrazine hydrate (10 eq) was added and the reaction was refluxed for 2 h. After completion of the reaction, the mixture was quenched by the addition of water (20 mL) and extracted with dichloromethane (3 × 20 mL). The combined organic layers were dried over anhydrous Na_2_SO_4_, filtered and concentrated under reduced pressure. The resulting products were purified by CCTLC in the Chromatothron as specified.

1-((3*R*,3a*S*)-*rel*-7-Methoxy-3-(4-methoxyphenyl)-6-nitro-3,3a,4,5-tetrahydro-2*H*-benzo[*g*]indazol-2-yl)ethan-1-one (**11a**) and 1-((3*R*,3a*R*)-*rel*-7-methoxy-3-(4-methoxyphenyl)-6-nitro-3,3a,4,5-tetrahydro-2*H*-benzo[*g*]indazol-2-yl)ethan-1-one (**11b**), following the general procedure B, compound **7** (150 mg, 0.45 mmol) reacted with hydrazine hydrate (220 μL, 4.5 mmol) in acetic acid (2.0 mL). The resulting products were purified by CCTLC in the Chromatothron (hexane:EtOAc 10:5). The fastest moving fractions provided 45 mg (26% yield) of **11a** while the next fractions provided **11b**. These later fractions required an additional purification by CCTLC in Chromatothron (CH_2_Cl_2_:MeOH, 10:0.1) to give 53 mg (30% yield) of **11b**. Both compounds were obtained as pale yellow solids. Experimental data for **11a**: Mp 107–109 °C. MS (ES, positive mode): *m*/*z* 396 [M + H]^+^. ^1^H-NMR (DMSO-*d_6_*, 400 MHz) δ: 1.91 (m, 1H, H-4), 2.09–2.36 (m, 4H, COCH_3_, H-4), 2.64 (m, 1H, H-5), 2.87 (m, 1H, H-5), 3.25 (ddd, *J* = 14.1, 9.7, 4.9 Hz, 1H, H-3a), 3.74 (s, 3H, OCH_3_), 3.93 (s, 3H, OCH_3_), 4.90 (d, *J* = 9.7 Hz, 1H, H-3), 6.89 (d, *J* = 8.7 Hz, 2H, H*_m_*), 7.20 (d, *J* = 8.7 Hz, 2H, H_o_), 7.35 (d, *J* = 8.9 Hz, 1H, H-8), 8.04 (d, *J* = 8.9 Hz, 1H, H-9). ^13^C-NMR (DMSO-*d_6_*, 100 MHz) δ: 22.1 (COCH_3_), 23.7 (C-5), 25.8 (C-4), 53.8 (C-3a), 55.1 (OCH_3_), 57.0 (OCH_3_), 66.3 (C-3), 112.2 (C-8), 113.9 (C_m_), 120.7, 127.2 (C_o_), 127.6 (C-9), 131.6, 133.8, 140.4, 151.3, 152.9, 158.3, 168.9 (C=O). Analytical HPLC (gradient 30–95% acetonitrile in 10 min): T_R_: 7.32; area: 98%. Anal. cal. for (C_21_H_21_N_3_O_5_): C, 63.79; H, 5.35, N, 10.63. Found: C,63.54; H, 5.38, N, 10.48. Experimental data for **11b**: Mp 215–217 °C. MS (ES, positive mode): *m*/*z* 396 [M + H]^+^. ^1^H-NMR (DMSO-*d_6_*, 400 MHz) δ: 0.76 (m, 1H, H-4), 1.72 (m, 1H, H-4), 2.32 (s, 3H, COCH_3_), 2.58 (m, 1H, H-5), 2.79 (m, 1H, H-5), 3.66–3.77 (m, 4H, OCH_3_, H-3a), 3.93 (s, 3H, OCH_3_), 5.62 (d, *J* = 11.0 Hz, 1H, H-3), 6.85 (d, *J* = 8.6 Hz, 2H, H*_m_*), 6.95 (d, *J* = 8.6 Hz, 2H, H_o_), 7.35 (d, *J* = 8.9 Hz, 1H, H-8), 8.14 (d, *J* = 8.9 Hz, 1H, H-9). ^13^C-NMR (DMSO-*d_6_*, 100 MHz) δ: 21.7 (COCH_3_), 22.6 (C-5), 23.7 (C-4), 46.9 (C-3a), 55.0 (OCH_3_), 57.0 (OCH_3_), 62.1 (C-3), 112.3 (C-8), 113.8 (C_m_), 120.6, 127.1 (C_o_), 127.8 (C-9), 129.1, 131.7, 140.3, 151.5, 153.4, 158.5, 166.9 (C=O). Analytical HPLC (gradient 30–95% acetonitrile in 10 min): T_R_: 5.58; area: 98%. Anal. cal. for (C_21_H_21_N_3_O_5_): C, 63.79; H, 5.35, N, 10.63. Found: C,63.60; H, 5.40, N, 10.49.

1-((3*R*,3a*S*)-*rel*-3-(3-Fluoro-4-methoxyphenyl)-7-methoxy-6-nitro-3,3a,4,5-tetrahydro-2*H*-benzo[*g*]indazol-2-yl)ethan-1-one (**12a**) and 1-((3*R*,3a*R*)-*rel*-3-(3-fluoro-4-methoxyphenyl)-7-methoxy-6-nitro-3,3a,4,5-tetrahydro-2*H*-benzo[*g*]indazol-2-yl)ethan-1-one (**12b**), following the general procedure B, compound **8** (200 mg, 0.56 mmol) reacted with hydrazine hydrate (280 μL, 5.8 mmol) in acetic acid (2.0 mL). The resulting products were purified by CCTLC in the Chromatothron (hexane:EtOAc 10:5). The fastest moving fractions provided 93 mg (40% yield) of **12a**, while the next fractions provided **12b**, which required an additional purification by CCTLC in Chromatothron (CH_2_Cl_2_:MeOH, 10:0.1) to give 80 mg (34% yield) of **12b**. Both compounds were obtained as pale yellow solids. Experimental data for **12a**: Mp 209–210 °C. MS (ES, positive mode): *m*/*z* 414 [M + H]^+^. ^1^H-NMR (DMSO-*d_6_*, 400 MHz) δ: 1.90 (m, 1H, H-4), 2.13–2.34 (m, 4H, COCH_3_, H-4), 2.64 (m, 1H, H-5), 2.87 (m, 1H, H-5), 3.28 (ddd, *J* = 14.8, 9.8, 4.9 Hz, 1H, H-3a), 3.82 (s, 3H, OCH_3_), 3.94 (s, 3H, OCH_3_), 4.91 (d, *J* = 9.8 Hz, 1H, H-3), 7.07 (dd, *J* = 8.5, 2.1 Hz, 1H, H_o’_), 7.10–7.17 (m, 2H, H*_o_*, H_m’_), 7.35 (d, *J* = 8.9 Hz, 1H, H-8), 8.04 (d, *J* = 8.9 Hz, 1H, H-9). ^13^C-NMR (DMSO-*d_6_*, 75 MHz) δ: 22.1 (COCH_3_), 23.6 (C-5), 25.7 (C-4), 53.6 (C-3a), 56.0 (OCH_3_), 57.0 (OCH_3_), 65.9 (C-3), 112.2 (C-8), 113.7 (d, ^2^*J_C-F_* = 19.0 Hz, C_o_), 113.8 (d, ^3^*J_C-F_* = 1.6 Hz, C_m’_), 120.6, 122.3 (d, ^4^*J_C-F_* = 3.5 Hz, C_o’_), 127.7 (C-9), 131.6, 134.7 (d, ^3^*J_C-F_* = 6.1 Hz, C-C3), 140.4, 146.1 (d, ^2^*J_C-F_* = 10.5 Hz, C-OCH_3_), 151.3, 151.4 (d, ^1^*J_C-F_* = 243.8 Hz, C-F), 153.0, 169.0 (C=O). Analytical HPLC (gradient 30–95% acetonitrile in 10 min): T_R_: 7.43; area: 97%. Anal. cal. for (C_21_H_20_FN_3_O_5_): C, 61.01; H, 4.88, N, 10.16. Found: C, 60.85; H, 4.93, N, 10.04. Experimental data for **12b**: Mp 127–129 °C. MS (ES, positive mode): *m*/*z* 414 [M + H]^+^. ^1^H-NMR (DMSO-*d_6_*, 400 MHz) δ: 0.79 (m, 1H, H-4), 1.77 (m, 1H, H-4), 2.33 (s, 3H, COCH_3_), 2.58 (m, 1H, H-5), 2.79 (m, 1H, H-5), 3.72 (ddd, *J* = 13.7, 11.0, 4.9 Hz, 1H, H-3a), 3.79 (s, 3H, OCH_3_), 3.93 (s, 3H, OCH_3_), 5.63 (d, *J* = 11.0 Hz, 1H, H-3), 6.80 (d, *J* = 8.3 Hz, 1H, H_o’_), 6.90 (dd, *J* = 12.2, 2.1 Hz, 1H, H*_o_*), 7.09 (t, *J* = 8.7 Hz, 1H, H_m’_), 7.35 (d, *J* = 9.0 Hz, 1H, H-8), 8.14 (d, *J* = 9.0 Hz, 1H, H-9). ^13^C-NMR (DMSO-*d_6_*, 75 MHz) δ: 21.6 (COCH_3_), 22.6 (C-5), 23.8 (C-4), 46.9 (C-3a), 56.0 (OCH_3_), 57.0 (OCH_3_), 61.6 (C-3), 112.3 (C-8), 113.7 (d, ^2^*J_C-F_* = 18.9 Hz, C_o_), 113.9 (d, ^3^*J_C-F_* = 0.6 Hz, C_m’_), 120.5, 122.0 (d, ^4^*J_C-F_* = 3.2 Hz, C_o’_), 127.9 (C-9), 130.1 (d, ^3^*J_C-F_* = 5.5 Hz, C-C3), 131.7, 140.3, 146.2 (d, ^2^*J_C-F_* = 10.5 Hz, C-OCH_3_), 151.3 (d, ^1^*J_C-F_* = 244.1 Hz, C-F), 151.6, 153.5, 167.0 (C=O). Analytical HPLC (gradient 30–95% acetonitrile in 10 min): T_R_: 6.93; area: 98%. Anal. cal. for (C_21_H_20_FN_3_O_5_.0.25H_2_0): C, 60.36; H, 4.94, N, 10.06. Found: C, 60.32; H, 4.88, N, 10.08.

1-((3*R*,3a*S*)-*rel*-7-Methoxy-3-(6-methoxypyridin-3-Yl)-6-nitro-3,3a,4,5-tetrahydro-2*H*-benzo[*g*]indazol-2-yl)ethan-1-one (**13a**) and 1-((3*R*,3a*R*)-*rel*-7-methoxy-3-(6-methoxypyridin-3-Yl)-6-nitro-3,3a,4,5-tetrahydro-2*H*-benzo[*g*]indazol-2-yl)ethan-1-one (**13b**), following the general procedure B, compound **9** (234 mg, 0.69 mmol) reacted with hydrazine hydrate (340 μL, 7.0 mmol) in acetic acid (2.0 mL). The resulting products were purified by CCTLC in the Chromatothron (hexane:EtOAc 10:5). The fastest moving fractions provided 104 mg (38% yield) of **13a**, while the next fractions provided **13b**, which required an additional purification by CCTLC in Chromatothron (CH_2_Cl_2_:MeOH, 10:0.2) to give 90 mg (33% yield) of **13b**. Both compounds were obtained as pale yellow solids. Experimental data for **13a**: Mp 208–210 °C. MS (ES, positive mode): *m*/*z* 397 [M + H]^+^. ^1^H-NMR (DMSO-*d_6_*, 400 MHz) δ: 1.92 (m, 1H, H-4), 2.17–2.30 (m, 4H, COCH_3_, H-4), 2.65 (m, 1H, H-5), 2.87 (m, 1H, H-5), 3.36 (ddd, *J* = 14.4, 10.0, 4.9 Hz, 1H, H-3a), 3.84 (s, 3H, OCH_3_), 3.94 (s, 3H, OCH_3_), 4.96 (d, *J* = 10.0 Hz, 1H, H-3), 6.79 (d, *J* = 8.6 Hz, 1H, H*_m_*), 7.36 (d, *J* = 8.9 Hz, 1H, H-8), 7.63 (dd, *J* = 8.6, 2.5 Hz, 1H, H_o_), 8.05 (d, *J* = 8.9 Hz, 1H, H-9), 8.12 (d, *J* = 2.5 Hz, 1H, H_o’_). ^13^C-NMR (DMSO-*d_6_*, 75 MHz) δ: 22.0 (COCH_3_), 23.7 (C-5), 25.5 (C-4), 53.1 (C-3a), 53.2 (OCH_3_), 57.0 (OCH_3_), 64.1 (C-3), 110.4, 112.3 (C-8), 120.6, 127.7 (C-9), 130.1, 131.7, 137.1, 140.4, 145.0, 151.4, 153.1, 162.8, 169.1 (C=O). Analytical HPLC (gradient 30–95% acetonitrile in 10 min): T_R_: 6.41; area: 96%. Anal. cal. for (C_20_H_20_N_4_O_5_): C, 60.60; H, 5.09, N, 14.13. Found: C, 60.42; H, 5.15, N, 14.02. Experimental data for **13b**: Mp 123–125 °C. MS (ES, positive mode): *m*/*z* 397 [M + H]^+^. ^1^H-NMR (DMSO-*d_6_*, 400 MHz) δ: 0.86 (m, 1H, H-4), 1.78 (m, 1H, H-4), 2.32 (s, 3H, COCH_3_), 2.60 (m, 1H, H-5), 2.81 (m, 1H, H-5), 3.76 (ddd, *J* = 13.6, 11.0, 4.9 Hz, 1H, H-3a), 3.81 (s, 3H, OCH_3_), 3.93 (s, 3H, OCH_3_), 5.68 (d, *J* = 11.0 Hz, 1H, H-3), 6.74 (d, *J* = 8.6 Hz, 1H, H*_m_*), 7.32 (dd, *J* = 8.6, 2.6 Hz, 1H, H_o_), 7.36 (d, *J* = 9.0 Hz, 1H, H-8), 7.92 (d, *J* = 2.5 Hz, 1H, H*_o’_*), 8.14 (d, *J* = 9.0 Hz, 1H, H-9). ^13^C-NMR (DMSO-*d_6_*, 75 MHz) δ: 21.6 (COCH_3_), 22.7 (C-5), 23.8 (C-4), 46.7 (C-3a), 53.1 (OCH_3_), 57.0 (OCH_3_), 59.9 (C-3), 110.4, 112.4 (C-8), 120.5, 125.8, 127.9 (C-9), 131.7, 137.1, 140.3, 144.6, 151.6, 153.6, 163.0, 167.0 (C=O). Analytical HPLC (gradient 30–95% acetonitrile in 10 min): T_R_: 5.98; area: 96%. Anal. cal. for (C_20_H_20_N_4_O_5_): C, 60.60; H, 5.09, N, 14.13. Found: C, 60.49; H, 5.18, N, 14.05.

1-((3*R*,3a*S*)-*rel*-7-Methoxy-6-nitro-3-(3,4,5-trimethoxyphenyl)-3,3a,4,5-tetrahydro-2*H*-benzo[*g*]indazol-2-yl)ethan-1-one (**14a**) and 1-((3*R*,3a*R*)-*rel*-7-methoxy-6-nitro-3-(3,4,5-trimethoxyphenyl)-3,3a,4,5-tetrahydro-2*H*-benzo[*g*]indazol-2-yl)ethan-1-one (**14b**), following the general procedure B, compound **10** (260 mg, 0.65 mmol) reacted with hydrazine hydrate (320 μL, 6.6 mmol) in acetic acid (2.0 mL). The resulting products were purified by CCTLC in the Chromatothron (CH_2_Cl_2_:EtOAc 20:1). The fastest moving fractions provided 55 mg (19 % yield) of **14a**, while the next fractions provided 108 mg (36% yield) of a mixture **14a** and **14b**, and the final fractions provided 32 mg (11 % yield) of **14b**. The mixture of diasteromers **14a** and **14b**, was submitted to two additional purification by CCTLC in Chromatothron using hexane:EtOAc (10:5) and CH_2_Cl_2_:MeOH (10:0.1), however both compounds were obtained in almost all collect fractions. The mixture **14a** and **14b** was used in the next step. Data for **14a** and **14b** were obtained from fractions containing a single diasteromer. Experimental data for **14a**: Mp 282–283 °C. MS (ES, positive mode): *m*/*z* 456 [M + H]^+^. ^1^H-NMR (DMSO-*d_6_*, 400 MHz) δ: 1.92 (m, 1H, H-4), 2.20–2.30 (m, 4H, COCH_3_, H-4), 2.65 (m, 1H, H-5), 2.87 (m, 1H, H-5), 3.29 (ddd, *J* = 13.2, 9.9, 4.9 Hz, 1H, H-3a), 3.64 (s, 3H, OCH_3_), 3.76 (s, 6H, OCH_3_), 3.94 (s, 3H, OCH_3_), 4.88 (d, *J* = 9.9 Hz, 1H, H-3), 6.57 (s, 2H, H_o_), 7.35 (d, *J* = 8.9 Hz, 1H, H-8), 8.06 (d, *J* = 8.9 Hz, 1H, H-9). ^13^C-NMR (DMSO-*d_6_*, 75 MHz) δ: 22.1 (COCH_3_), 23.6 (C-5), 25.8 (C-4), 53.7 (C-3a), 55.9 (OCH_3_), 57.0 (OCH_3_), 59.9 (OCH_3_), 67.1 (C-3), 103.1 (C-8), 112.2, 120.7, 127.7 (C-9), 131.7, 136.3, 137.7, 140.4, 151.3, 152.9, 153.0, 169.1 (C=O). Analytical HPLC (gradient 30–95% acetonitrile in 10 min): T_R_: 6.66; area: 98%. Anal. cal. for (C_23_H_25_N_3_O_7_): C, 60.65; H, 5.53, N, 9.23. Found: C, 60.35; H, 5.51, N, 8.99. Experimental data for **14b**: Mp 270–272 °C. MS (ES, positive mode): *m*/*z* 456 [M + H]^+^. ^1^H-NMR (DMSO-*d_6_*, 400 MHz) δ: 0.84 (m, 1H, H-4), 1.83 (m, 1H, H-4), 2.35 (s, 3H, COCH_3_), 2.61 (m, 1H, H-5), 2.80 (m, 1H, H-5), 3.61 (s, 3H, OCH_3_), 3.65–3.81 (m, 7H, H-3a, OCH_3_), 3.93 (s, 3H, OCH_3_), 5.62 (d, *J* = 11.1 Hz, 1H, H-3), 6.31 (s, 2H, H*_o_*), 7.34 (d, *J* = 8.9 Hz, 1H, H-8), 8.14 (d, *J* = 8.9 Hz, 1H, H-9). ^13^C-NMR (DMSO-*d_6_*, 75 MHz) δ: 21.6 (COCH_3_), 22.5 (C-5), 23.8 (C-4), 46.9 (C-3a), 55.8 (OCH_3_), 57.0 (OCH_3_), 59.9 (OCH_3_), 62.6 (C-3), 103.0, 112.3 (C-8), 120.6, 127.9, 131.8, 133.0, 136.5, 140.3, 151.5, 152.9, 153.7, 167.1 (C=O). Analytical HPLC (gradient 30-95% acetonitrile in 10 min): T_R_: 6.18; area: 96%. Anal. cal. for (C_23_H_25_N_3_O_7_): C, 60.65; H, 5.53, N, 9.23. Found: C, 60.39; H, 5.62, N, 9.12.

#### 3.2.4. General Procedure for the Synthesis of Pyrazolines Bearing 6-Amino-7-Methoxy-3,3a,4,5-Tetrahydro-*2H*-Benzo[g]indazole moieties **15**–**18a,b** (General Procedure C)

To a suspension containing the appropriate nitro compound (1.0 eq) and iron powder (10 eq) in ethanol-water (2:1, 3.0 mL), one drop of 37% HCl was added. The reaction mixture was heated to reflux for 1 h. EtOAc (10 mL) was added to the mixture, washed with water and the aqueous phase was further extracted with EtOAc (3 × 10 mL). The combined organic layers were dried over anhydrous Na_2_SO_4_, filtered and evaporated under reduced pressure. The residue was purified by CCTLC in the Chromatothron as specified.

1-((3*R*,3a*S*)-*rel*-6-Amino-7-methoxy-3-(4-methoxyphenyl)-3,3a,4,5-tetrahydro-2*H*-tenzo[*g*]indazol-2-yl)ethan-1-one (**15a**), following the general procedure C, compound **11a** (60 mg, 0.15 mmol) reacted with iron powder (88 mg, 1.6 mmol) in EtOH:H_2_O (2:1, 3.0 mL). The crude product was purified by CCTLC in the Chromatothron (CH_2_Cl_2_:EtOAc, 10:2) to provide 40 mg (73% yield) of **15a**, as a beige solid. Mp 219–220 °C. MS (ES, positive mode): *m*/*z* 366 [M + H]^+^. ^1^H-NMR (DMSO-*d_6_*, 400 MHz) δ: 1.83 (m, 1H, H-4), 2.15–2.27 (m, 4H, COCH_3_, H-4), 2.45 (m, 1H, H-5), 2.74 (m, 1H, H-5), 3.05 (ddd, *J* = 13.8, 9.2, 4.8 Hz, 1H, H-3a), 3.74 (s, 3H, OCH_3_), 3.82 (s, 3H, OCH_3_), 4.61 (br s, 2H, NH_2_), 4.83 (d, *J* = 9.2 Hz, 1H, H-3), 6.85 (d, *J* = 8.5 Hz, 1H, H-8), 6.89 (d, *J* = 8.6 Hz, 2H, H*_m_*), 7.18 (d, *J* = 8.6 Hz, 2H, H_o_), 7.19 (d, *J* = 8.5 Hz, 1H, H-9). ^13^C-NMR (DMSO-*d_6_*, 75 MHz) δ: 22.1 (COCH_3_), 23.8 (C-5), 26.8 (C-4), 54.7 (C-3a), 55.1 (OCH_3_), 55.6 (OCH_3_), 66.0 (C-3), 108.8 (C-8), 112.8 (C-9), 113.9 (C_m_), 119.9, 123.4, 127.0 (C_o_), 134.4, 134.7, 147.5, 155.6, 158.2, 168.4 (C=O). Analytical HPLC (gradient 30–95% acetonitrile in 10 min): T_R_: 5.12; area: 97%. Anal. cal. for (C_21_H_23_N_3_O_3_): C, 69.02; H, 6.34, N, 11.50. Found: C, 68.70; H, 6.47, N, 11.21.

1-((3*R*,3a*R*)-*rel*-6-Amino-7-methoxy-3-(4-methoxyphenyl)-3,3a,4,5-tetrahydro-2*H*-benzo[*g*]indazol-2-yl)ethan-1-one (**15b**), following the general procedure C, compound **11b** (71 mg, 0.18 mmol) reacted with iron powder (107 mg, 1.9 mmol) in EtOH:H_2_O (2:1, 3.0 mL). The crude product was purified by CCTLC in the Chromatothron (CH_2_Cl_2_:EtOAc, 10:2) to provide 49 mg (75% yield) of **15b**, as a white solid. Mp 217–219 °C. MS (ES, positive mode): *m*/*z* 366 [M + H]^+^. ^1^H-NMR (DMSO-*d_6_*, 400 MHz) δ: 0.72 (m, 1H, H-4), 1.72 (m, 1H, H-4), 2.28 (s, 3H, COCH_3_), 2.40 (m, 1H, H-5), 2.68 (m, 1H, H-5), 3.57 (ddd, *J* = 15.2, 10.8, 4.7 Hz, 1H, H-3a), 3.71 (s, 3H, OCH_3_), 3.81 (s, 3H, OCH_3_), 4.58 (br s, 2H, NH_2_), 5.55 (d, *J* = 10.8 Hz, 1H, H-3), 6.82–6.88 (m, 3H, H*_m_*, H-8), 6.96 (d, *J* = 8.3 Hz, 2H, H*_o_*), 7.31 (d, *J* = 8.4 Hz, 1H, H-9). ^13^C-NMR (DMSO-*d_6_*, 75 MHz) δ: 21.7 (COCH_3_), 23.3 (C-5), 23.6 (C-4), 47.3 (C-3a), 55.0 (OCH_3_), 55.6 (OCH_3_), 61.8 (C-3), 108.9 (C-8), 113.1 (C-9), 113.7 (C_m_), 119.8, 123.7, 127.1 (C_o_), 129.6, 134.7, 147.7, 156.2, 158.3, 166.4 (C=O). Analytical HPLC (gradient 30–95% acetonitrile in 10 min): T_R_: 4.22; area: 98%. Anal. cal. for (C_21_H_23_N_3_O_3_): C, 69.02; H, 6.34, N, 11.50. Found: C, 68.64; H, 6.53, N, 11.04.

1-((3*R*,3a*S*)-*rel*-6-Amino-3-(3-fluoro-4-methoxyphenyl)-7-methoxy-3,3a,4,5-tetrahydro-2*H*-benzo[*g*]indazol-2-yl)ethan-1-one (**16a**), following the general procedure C, compound **12a** (69 mg, 0.17 mmol) reacted with iron powder (102 mg, 1.8 mmol) in EtOH:H_2_O (2:1, 3.0 mL). The crude product was purified by CCTLC in the Chromatothron (CH_2_Cl_2_:EtOAc, 10:2) to provide 43 mg (67% yield) of **16a**, as a white solid. Mp 154–156 °C. MS (ES, positive mode): *m*/*z* 384 [M + H]^+^. ^1^H-NMR (DMSO-*d_6_*, 400 MHz) δ: 1.82 (m, 1H, H-4), 2.15–2.35 (m, 4H, COCH_3_, H-4), 2.45 (m, 1H, H-5), 2.74 (m, 1H, H-5), 3.09 (ddd, *J* = 13.9, 9.4, 4.8 Hz, 1H, H-3a), 3.82 (s, 6H, OCH_3_), 4.61 (br s, 2H, NH_2_), 4.84 (d, *J* = 9.4 Hz, 1H, H-3), 6.85 (d, *J* = 8.5 Hz, 1H, H-8), 7.05 (dd, *J* = 8.5, 2.1 Hz, 1H, H_o’_), 7.09–7.14 (m, 2H, H*_o_*, H*_m’_*), 7.20 (d, *J* = 8.4 Hz, 1H, H-9). ^13^C-NMR (DMSO-*d_6_*, 75 MHz) δ: 22.1 (COCH_3_), 23.8 (C-5), 26.7 (C-4), 54.4 (C-3a), 55.6 (OCH_3_), 56.0 (OCH_3_), 65.6 (C-3), 108.8 (C-8), 112.8 (C-9), 113.5 (d, ^2^*J_C-F_* = 18.7 Hz, C_o_), 113.9 (d, ^3^*J_C-F_* = 1.2 Hz, C_m’_), 119.8, 122.0 (d, ^4^*J_C-F_* = 3.4 Hz, C_o’_), 123.5, 134.8, 135.2 (d, ^3^*J_C-F_* = 6.0 Hz, C-C3), 146.0 (d, ^2^*J_C-F_* = 10.6 Hz, C-OCH_3_), 147.6, 151.4 (d, ^1^*J_C-F_* = 243.6 Hz, C-F), 155.7, 168.6 (C=O). Analytical HPLC (gradient 30–95% acetonitrile in 10 min): T_R_: 5.13; area: 97%. Anal. cal. for (C_21_H_22F_N_3_O_3_): C, 65.78; H, 5.78, N, 10.96. Found: C, 65.82; H, 5.89, N, 10.78.

1-((3*R*,3a*R*)-*rel*-6-Amino-3-(3-fluoro-4-methoxyphenyl)-7-methoxy-3,3a,4,5-tetrahydro-2*H*-benzo[*g*]indazol-2-yl)ethan-1-one (**16b**), following the general procedure C, compound **12b** (50 mg, 0.12 mmol) reacted with iron powder (67 mg, 1.2 mmol) in EtOH:H_2_O (2:1, 3.0 mL). The crude product was purified by CCTLC in the Chromatothron (CH_2_Cl_2_:EtOAc, 10:2) to provide 27 mg (59% yield) of **16b**, as a white solid. Mp 197–199 °C. MS (ES, positive mode): *m*/*z* 384 [M + H]^+^. ^1^H-NMR (DMSO-*d_6_*, 400 MHz) δ: 0.73 (m, 1H, H-4), 1.77 (m, 1H, H-4), 2.29 (s, 3H, COCH_3_), 2.42 (m, 1H, H-5), 2.70 (m, 1H, H-5), 3.59 (ddd, *J* = 13.8, 10.9, 4.9 Hz, 1H, H-3a), 3.79 (s, 3H, OCH_3_), 3.81 (s, 3H, OCH_3_), 4.59 (br s, 2H, NH_2_), 5.56 (d, *J* = 10.9 Hz, 1H, H-3), 6.80 (d, *J* = 8.5 Hz, 1H, H*_o_*), 6.85 (d, *J* = 8.6 Hz, 1H, H-8), 6.89 (dd, *J* = 8.5, 2.1 Hz, 1H, H_o’_), 7.09 (t, *J* = 8.7 Hz, 1H, H_m’_), 7.31 (d, *J* = 8.6 Hz, 1H, H-9). ^13^C-NMR (DMSO-*d_6_*, 75 MHz) δ: 21.6 (COCH_3_), 23.2 (C-5), 23.6 (C-4), 47.3 (C-3a), 55.6 (OCH_3_), 56.0 (OCH_3_), 61.3 (C-3), 108.9 (C-8), 113.1 (C-9), 113.7 (d, ^2^*J_C-F_* = 18.9 Hz, C_o_), 113.8 (C_m’_), 119.7, 122.0 (d, ^4^*J_C-F_* = 3.1 Hz, C_o’_), 123.6, 130.7 (d, ^3^*J_C-F_* = 5.5 Hz, C-C3), 134.7, 146.1 (d, ^2^*J_C-F_* = 10.5 Hz, C-OCH_3_), 147.7, 151.3 (d, ^1^*J_C-F_* = 244.2 Hz, C-F), 156.2, 166.5 (C=O). Analytical HPLC (gradient 30-95% acetonitrile in 10 min): T_R_: 4.35; area: 95%). Anal. cal. for (C_21_H_22F_N_3_O_3_): C, 65.78; H, 5.78, N, 10.96. Found: C, 65.87; H, 5.91, N, 10.82.

1-((3*R*,3a*S*)-*rel*-6-Amino-7-methoxy-3-(6-methoxypyridin-3-yl)-3,3a,4,5-tetrahydro-2*H*-benzo[*g*]indazol-2-yl)ethan-1-one (**17a**), following the general procedure C, compound **13a** (63 mg, 0.16 mmol) reacted with iron powder (90 mg, 1.6 mmol) in EtOH:H_2_O (2:1, 3 mL). The crude product was purified by CCTLC in the Chromatothron (CH_2_Cl_2_:EtOAc, 10:4) to provide 30 mg (52% yield) of **17a**, as a white solid. Mp 185–187 °C. MS (ES, positive mode): *m*/*z* 367 [M + H]^+^. ^1^H-NMR (DMSO-*d_6_*, 400 MHz δ 1.83 (m, 1H, H-4), 2.16–2.31 (m, 4H, COCH_3_, H-4), 2.45 (m, 1H, H-5), 2.75 (m, 1H, H-5), 3.16 (ddd, *J* = 13.3, 9.6, 4.8 Hz, 1H, H-3), 3.82 (s, 3H, OCH_3_), 3.84 (s, 3H, OCH_3_), 4.62 (br s, 2H, NH_2_), 4.88 (d, *J* = 9.6 Hz, 1H, H-3a), 6.78 (d, *J* = 8.6 Hz, 1H, H*_m_*), 6.85 (d, *J* = 8.5 Hz, 1H, H-8), 7.21 (d, *J* = 8.5 Hz, 1H, H-9), 7.60 (dd, *J* = 8.6, 2.5 Hz, 1H, H_o_), 8.11 (d, *J* = 2.5 Hz, 1H, H_o’_). ^13^C-NMR (DMSO-*d_6_*, 75 MHz) δ: 22.1 (COCH_3_), 23.8 (C-5), 26.5 (C-4), 53.2 (OCH_3_), 53.9 (C-3a), 55.6 (OCH_3_), 63.9 (C-3), 108.8 (C-8), 110.4 (C*_m_*), 112.9 (C-9), 119.7, 123.5, 130.6, 134.8, 137.0 (C_o_), 144.8 (C_o’_), 147.6, 155.8, 162.7, 168.7 (C=O). Analytical HPLC (gradient 30-95% acetonitrile in 10 min): T_R_: 2.67; area: 98%. Anal. cal. for (C_20_H_22_N_4_O_3_): C, 65.56; H, 6.05; N, 15.29. Found: C, 65.13; H, 6.09; N, 15.00.

1-((3*R*,3a*R*)-*rel*-6-Amino-7-methoxy-3-(6-methoxypyridin-3-yl)-3,3a,4,5-tetrahydro-2*H*-benzo[*g*]indazol-2-yl)ethan-1-one (**17b**), following the general procedure C, compound **13b** (65 mg, 0.16 mmol) reacted with iron powder (97 mg, 1.7 mmol) in EtOH:H_2_O (2:1, 3.0 mL). The crude product was purified by CCTLC in the Chromatothron (CH_2_Cl_2_:EtOAc, 10:4) to provide 29 mg (50% yield) of **17b**, as a white solid. Mp 202–204 °C. MS (ES, positive mode): *m*/*z* 367 [M + H]^+^. ^1^H-NMR (DMSO-*d_6_*, 400 MHz) δ: 0.80 (m, 1H, H-4), 1.77 (m, 1H, H-4), 2.28 (s, 3H, COCH_3_), 2.43 (m, 1H, H-5), 2.71 (m, 1H, H-5), 3.63 (ddd, *J* = 15.0, 11.0, 4.7 Hz, 1H, H-3a), 3.81 (s, 3H, OCH_3_), 3.82 (s, 3H, OCH_3_), 4.60 (br s, 2H, NH_2_), 5.61 (d, *J* = 11.0 Hz, 1H), 6.74 (d, *J* = 8.5 Hz, 1H, H*_m_*), 6.86 (d, *J* = 8.5 Hz, 1H, H-8), 7.26–7.35 (m, 2H, H-9, H_o_), 7.91 (d, *J* = 2.5 Hz, 1H, H_o’_). ^13^C-NMR (DMSO-*d_6_*, 75 MHz) δ: 21.7 (COCH_3_), 23.5 (C-5), 23.7 (C-4), 47.3 (C-3a), 53.2 (OCH_3_), 55.7 (OCH_3_), 59.6 (C-3), 108.9 (C-8), 110.3 (C_m_), 113.2 (C-9), 119.7, 123.7, 126.3, 134.7, 137.2 (C_o_), 144.6 (C_o’_), 147.8, 156.4, 162.9, 166.7 (C=O). Analytical HPLC (gradient 30–95% acetonitrile in 10 min): T_R_: 2.08; area: 96%. Anal. cal. for (C_20_H_22_N_4_O_3_): C, 65.56; H, 6.05; N, 15.29. Found: C, 65.24; H, 6.12; N, 15.07.

1-((3*R*,3a*S*)-*rel*-6-Amino-7-methoxy-3-(3,4,5-trimethoxyphenyl)-3,3a,4,5-tetrahydro-2*H*-benzo[*g*]indazol-2-yl)ethan-1-one (**18a**) and 1-((3*R*,3a*R*)-*rel*-6-amino-7-methoxy-3-(3,4,5-trimethoxy- phenyl)-3,3a,4,5-tetrahydro-2*h*-benzo[*g*]indazol-2-yl)Ethan-1-One (**18b**), following the general procedure C, a mixture of compounds **14a** and **14b** (74 mg, 0.16 mmol) reacted with iron powder (100 mg, 1.8 mmol) in EtOH:H_2_O (2:1, 3.0 mL). The crude product was purified by CCTLC in the Chromatothron (CH_2_Cl_2_:EtOAc, 10:2). The fastest moving fractions afforded 17 mg (25% yield) of **18a**, while the slowest moving fractions provided 30 mg (43% yield) of **18b**. Both compounds were obtained as white solids. Experimental data for **18a**: Mp 225–227 °C. MS (ES, positive mode): *m*/*z* 426 [M + H]^+^. ^1^H-NMR (DMSO-*d_6_*, 400 MHz) δ: 1.83 (m, 1H, H-4), 2.23–2.32 (m, 4H, COCH_3_, H-4), 2.47 (m, 1H, H-5), 2.75 (m, 1H, H-5), 3.11 (ddd, *J* = 14.0, 9.5, 4.9 Hz, 1H, H-3a), 3.64 (s, 3H, OCH_3_), 3.76 (s, 6H, OCH_3_), 3.82 (s, 3H, OCH_3_), 4.62 (br s, 2H, NH_2_), 4.80 (d, *J* = 9.5 Hz, 1H, H-3), 6.54 (s, 2H, H_o_), 6.85 (d, *J* = 8.5 Hz, 1H, H-8), 7.21 (d, *J* = 8.5 Hz, 1H, H-9). ^13^C-NMR (DMSO-*d_6_*, 75 MHz) δ: 22.1 (COCH_3_), 23.8 (C-5), 26.8 (C-4), 54.5 (C-3a), 55.6 (OCH_3_), 55.8 (OCH_3_), 59.9 (OCH_3_), 66.8 (C-3), 102.8 (C*_o_*), 108.8 (C-8), 112.8 (C-9), 119.8, 123.5, 134.8, 136.2, 138.2, 147.5, 153.0, 155.6, 168.7 (C=O). Analytical HPLC (gradient 30–95% acetonitrile in 10 min): T_R_: 4.67; area: 95%. Anal. cal. for (C_23_H_27_N_3_O_5_): C, 64.93; H, 6.40; N, 9.88. Found: C, 64.81; H, 6.55; N, 9.85. Experimental data for **18b**: Mp 181–183 °C. MS (ES, positive mode): *m*/*z* 426 (M+H)^+^. ^1^H-NMR (DMSO-*d_6_*, 400 MHz) δ: 0.78 (m, 1H, H-4), 1.83 (m, 1H, H-4), 2.32 (s, 3H, COCH_3_), 2.41 (m, 1H, H-5), 2.70 (m, 1H, H-5), 3.55 – 3.67 (m, 4H, H-3a, OCH_3_), 3.71 (s, 6H, OCH_3_), 3.82 (s, 3H, OCH_3_), 4.59 (br s, 2H, NH_2_), 5.56 (d, *J* = 11.0 Hz, 1H, H-3), 6.32 (s, 2H, H*_o_*), 6.85 (d, *J* = 8.6 Hz, 1H, H-8), 7.32 (d, *J* = 8.6 Hz, 1H, H-9). ^13^C-NMR (DMSO-*d_6_*, 75 MHz) δ: 21.6 (COCH_3_), 23.1 (C-5), 23.7 (C-4), 47.3 (C-3a), 55.6 (OCH_3_), 55.8 (OCH_3_), 59.9 (OCH_3_), 62.3 (C-3), 103.1 (C_o_), 108.8 (C-8), 113.1, (C-9) 119.8, 123.7, 133.5, 134.7, 136.4, 147.7, 152.8, 156.4, 166.6 (C=O). Analytical HPLC (gradient 30–95% acetonitrile in 10 min): T_R_: 3.62; area: 97%. Anal. cal. for (C_23_H_27_N_3_O_5_): C, 64.93; H, 6.40; N, 9.88. Found: C, 64.79; H, 6.52; N, 9.81.

### 3.3. Antiproliferative Activity

The tumor cell lines were acquired from the American Type Culture Collection (ATCC, Manassas, VA, USA), except for the DND-41 cell line, which was purchased from the Deutsche Sammlung von Mikroorganismen und Zellkulturen (DSMZ Leibniz-Institut, Germany), and the Hap-1 cell line was ordered from Horizon Discovery (Horizon Discovery Group, Waterbeach, UK). All cell lines were cultured as recommended by the suppliers. Media were purchased from Gibco Life Technologies (Waltham, MA, USA), and supplemented with 10% fetal bovine serum (HyClone, GE Healthcare Life Sciences, Chicago, IL, USA). Adherent cell lines HCT-116, NCI-H460, Hap-1 and Capan-1 cells were seeded at a density between 500 and 1500 cells per well, in 384-well, black walled, clear-bottomed tissue culture plates (Greiner Bio-One, Kremsmünster, Austria). After overnight incubation, cells were treated with the test compounds at seven different concentrations ranging from 100 to 6.4 × 10^−3^ µM. Suspension cell lines HL-60, K-562 and DND-41 were seeded at densities ranging from 2500 to 5500 cells per well in 384-well, black walled, clear-bottomed tissue culture plates containing the test compounds at the same seven concentration points.

The plates were incubated and monitored at 37 °C for 72 h in an IncuCyte^®^ (Essen BioScience Inc., Ann Arbor, MI, USA) for real-time imaging of cell proliferation. Images were taken every 3 h, with one field imaged per well under 10× magnification. Area under the curve (AUC) values were calculated and used to determine the IC_50_ values.

### 3.4. Antibacterial Activity

The nitro compounds were tested to determine the antibacterial activity against gram-negative and gram-positive bacteria. Wild-type and multi-resistant strains were included as follows: methicillin-susceptible *Staphylococcus aureus* ATCC 25923 (MSSA), methicillin-resistant *Staphylococcus aureus* ATCC 43300 (MRSA), vancomycin-intermediate *Staphylococcus aureus* (VISA), *Escherichia coli* ATCC 25922, carbapenemase–positive *Klebsiella pneumoniae* BAA 1705, *Klebsiella pneumoniae* ATCC 700603 (extended spectrum beta lactamase, ESBL positive), *Pseudomonas aeruginosa* ATCC 27853 and *Neisseria gonorrhoeae* ATCC 49226. Stock solutions (100 mg/mL) of the compounds were prepared in dimethyl sulfoxide (DMSO) and diluted to a final screening concentration of 1 mg/mL. An initial screening of bacterial inhibition was performed by agar diffusion method. Briefly, sterile Mueller Hinton agar (MHA, BBL) was prepared in Petri dishes and inoculated with a bacterial suspension prepared in trypticase soy broth and adjusted to 1.5 × 10^8^ colony forming unit CFU/mL (i.e., 0.08–0.1 OD at 600 nm) [33]. Wells (6 mm in diameter) were punched in the agar and 10 µL of each compound (stock solution) was filled into each well. Dimethyl sulfoxide and trypticase soy broth were included as negative controls (i.e., no inhibition of bacterial growth). Gentamicin and tetracycline (Sigma-Aldrich) were included as positive controls of growth inhibition. Compounds showing growth inhibition were tested at least twice before being selected for microdilution testing. For *N*. *gonorrhoeae* the agar diffusion method was also used in the screening process with some modifications. For this method, 200 µL of a bacterial suspension (1.5 × 10^8^ CFU/mL) was inoculated in gonococcal (GC) agar (BBL) supplemented with 1% isovitalex (BBL), then the compounds were added to the wells as above mentioned and incubated at 35–36.5 °C in 5% CO_2_ atmosphere for 48 h. Penicillin, ceftriaxone and ciprofloxacin (BBL) were used as controls [34].

#### 3.4.1. Microdilution Test

Minimum inhibitory concentration (MIC) was determined in those compounds with reproducible bacterial growth inhibition at the screening. Bacterial suspensions were adjusted with Mueller Hinton broth (MHB) to a concentration of 5 × 10^5^–8 × 10^5^ [33]. Stock solutions of the newly synthesized compounds were diluted in MHB containing 5% DMSO and 0.1% Tween 80 [35] and added to 90 µL of the bacterial inoculum. The microplates were incubated for 24 h at 35–37 °C. MICs were defined as the lowest concentration with visible inhibition of bacterial growth [33] and/or detection using resazurin (1 mg/mL) using a microplate spectrophotometer (Cytation 3M, Biotek, Winooski, VT, USA). Gentamicin (Sigma-Aldrich, St. Louis, MO, USA) and tetracycline were included as controls of growth inhibition; MHB and DMSO were used as a negative control. Experiments were performed in duplicate and replicated at least three times.

For *N*. *gonorrhoeae*, those compounds with visible growth inhibition in the screening test were further tested for MIC on agar plates as described by the Centers for Disease Control and Prevention [36] and the Clinical and Laboratory Standards Institute [34] with modifications. Briefly, GC agar supplemented with 1% isovitalex was prepared to contain increasing concentrations of the compounds and inoculated with 5 µL of the bacterial suspension (i.e., 1 × 10^4^ CFU) [36]. The lowest concentration of the compound that inhibited bacterial growth was determined as the MIC. Bacterial growth was examined and verified using the oxidase test. Experiments were performed in duplicate and replicated at least three times.

#### 3.4.2. Hemolytic Activity

The ability to induce hemolysis was evaluated to compounds that showed antibacterial activity following the method of cytotoxicity by spectrophotometry. The method was adapted from Conceição et al. [37] with modifications. Briefly, 240 μL of Human Red blood cells (huRBC) adjusted to 5% hematocrit in phosphate buffer saline (PBS) were placed into each well of 96-well plate and subsequently exposed to 200 µg/mL of the selected compounds (i.e., 10 μL of 5 mg/mL working solution of each compound in MHB with 5% DMSO 0.1% Tween-80). As positive control for hemolytic activity 10 μL SDS 1% was added. For a negative control only medium with no chemicals was added to huRBC. Free hemoglobin was measured after 24 h incubation at 37 °C by spectrophotometry (420 nm Cytation 3M, Biotek, Winooski, VT, USA). Non-specific absorbance was subtracted from a blank. Determinations were done by triplicate in at least two independent experiments.

## 4. Conclusions

A series of new benzo[*g*]indazole compounds **11**–**18a,b** incorporating 6-nitro and 6-amino groups were synthesized by cyclocondensation of 2-benzylidene-1-tetralones **7**–**10** with hydrazine hydrate in presence of acetic acid, resulting in the formation of *cis* (3*R*,3a*R-rel*) and *trans* (3*R*,3a*S-rel*) diastereoisomeric pyrazoline derivatives. The stereochemical assignment of the obtained diasteroisomers was performed based on experimental NMR data and a convenient criterion concerning the chemical shift of the H-4 protons has been proposed for the assignment of both isomers. The antiproliferative evaluation against a panel of tumor cell lines revealed that the 6-nitro benzo[*g*]indazoles **11a**, **11b**, **12a** and **12b** display IC_50_ values between 5–15 µM against NCI-H460 (lung carcinoma) while they were less active against other cell types. The *in vitro* screening for antibacterial activity showed that compounds **12a** and **13b** exhibited antigonococcal activity by inhibiting bacterial growth at MIC values of 250 and 62.5 μg/mL, respectively. None of the active compounds (**12a** and **13b**) showed hemolytic activity suggesting low membrane interactions and toxicity.

## Figures and Tables

**Table 1:** Antiproliferative activity of benzo[*g*]indazole derivatives expressed in IC_50_^a^ (μM).

Comp.	Capan-1	HCT-116	NCI-H460	DND-41	HL-60	K-562	Z-138
**11a**	40.8 ± 5.0	29.9 ± 9.0	10.8 ± 1.6	>100	30.9 ± 14.8	46.5 ± 0.2	>100
**11b**	27.0 ± 4.0	25.3 ± 18.9	11.8 ± 1.6	>100	38.0 ± 10.1	34.5 ± 5.9	70.4 ± 14.8
**12a**	42.9 ± 1.6	26.9 ± 4.8	5.4 ± 0.5	>100	42.5 ± 0.5	64.3 ± 17.9	49.7 ± 25.2
**12b**	17.9 ± 3.5	36.0 ± 6.8	14.9 ± 2.9	>100	38.9 ± 2.2	59.7 ± 4.8	45.1 ± 27.5
**13a**	73.4 ± 26.6	36.5 ± 1.2	30.9 ± 10.0	51.0 ± 7.6	42.7 ± 3.9	73.1 ± 3.1	39.7 ± 6.9
**13b**	32.3 ± 11.1	48.9 ± 6.8	45.0 ± 1.7	54.4 ± 10.2	42.9 ± 1.9	77.6 ± 9.1	42.3 ± 8.4
**14a**	57.8 ± 2.4	82.0 ± 9.0	43.7 ± 4.7	>100	28.6 ± 35.7	>100	>100
**14b**	42.9 ± 12.3	>100	44.6 ± 0.8	>100	26.8 ± 36.6	>100	>100
**15a**	45.1 ± 20.6	43.5 ± 6.0	42.1 ± 7.5	38.6 ± 7.2	55.9 ± 20.8	51.0 ± 29.2	43.9 ± 28.1
**15b**	53.5 ± 18.8	>100	54.1 ± 4.6	50.1 ± 25.0	44.8 ± 27.6	85.9 ± 7.1	50.1 ± 25.0
**16a**	36.7 ± 14.4	45.0 ± 11.5	39.3 ± 8.5	30.3 ± 7.4	38.7 ± 7.5	44.9 ± 21.8	50.2 ± 0.9
**16b**	42.6 ± 3.9	66.1 ± 31.3	33.7 ± 10.88	37.7 ± 6.4	65.9 ± 20.2	75.7 ± 21.0	48.0 ± 26.0
**17a**	56.4 ± 11.6	62.2 ± 10.9	47.3 ± 15.3	42.8 ± 28.6	49.0 ± 25.5	>100	>100
**17b**	60.7 ± 1.7	80.6 ± 9.7	43.9 ± 9.6	66.1 ± 18.6	65.8 ± 17.1	>100	>100
**18a**	30.3 ± 9.0	45.2 ± 2.6	36.6 ± 10.4	50.6 ± 8.5	52.5 ± 16.3	>100	89.9 ± 5.0
**18b**	32.7 ± 22.0	52.1 ± 15.3	44.4 ± 2.2	40.8 ± 3.3	41.6 ± 5.0	69.2 ± 4.3	60.1 ± 1.8
Docetaxel	0.0042 ± 0.0021	0.0009 ± 0.0008	0.0038 ± 0.0029	0.0033 ± 0.0014	0.0023 ± 0.0003	0.0037 ± 0.0003	0.0011 ± 0.0008
Stauroporine	0.0007 ± 0.0002	0.00010 ± 0.0000	0.0015 ± 0.0004	0.0043 ± 0.0022	0.0074 ± 0.0017	0.0224 ± 0.0074	0.0003 ± 0.0001

Capan-1: pancreatic adenocarcinoma; Hap1: chronic myeloid leukemia; HCT-116: colorectal carcinoma; NCI-H460: lung carcinoma; DND-41: acute lymphoblastic leukemia; HL-60: acute myeloid leukemia; K-562: chronic myeloid leukemia; Z-138: non-Hodgkin lymphoma. ^a^ IC_50_: Concentration of each compound that inhibits 50% of cell proliferation. Mean value of two experiments ±SD.

**Table 2:** In vitro antibacterial activity of nitro derivatives expressed in MIC ^a^ (μg/mL).

Comp.	*S. aureus* ATCC 25923	*S. aureus* ATCC 43300	*VISA*	*E. coli* ATCC 25922	*P. aeruginosa* ATCC 27853	*K. pneumoniae* ATCC700603	*K. pneumoniae* BAA 1705	*N. gonorrhoeae* ATCC 49226
**7**	>1000	>1000	>1000	>1000	>1000	>1000	>1000	≥500
**8**	>1000	>1000	>1000	>1000	>1000	>1000	>1000	>1000
**9**	>1000	>1000	>1000	>1000	>1000	>1000	>1000	>1000
**10**	>1000	>1000	>1000	>1000	>1000	>1000	>1000	>1000
**11a**	>1000	>1000	>1000	>1000	>1000	>1000	>1000	≥500
**11b**	>1000	>1000	>1000	>1000	>1000	>1000	>1000	≥500
**12a**	>1000	>1000	>1000	>1000	>1000	>1000	>1000	250
**12b**	>1000	>1000	>1000	>1000	>1000	>1000	>1000	>1000
**13a**	>1000	>1000	>1000	>1000	>1000	>1000	>1000	≥500
**13b**	>1000	>1000	>1000	>1000	>1000	>1000	>1000	62.5
**14a**	>1000	>1000	>1000	>1000	>1000	>1000	>1000	≥500
**14b**	>1000	>1000	>1000	>1000	>1000	>1000	>1000	≥500
GEN ^b^	<1	<0.25	<0.25	<0.625	1	0.625	<0.312	1.25–2.5
TET ^c^								1

^a^ MIC: Minimum inhibitory concentration of each compound that inhibit visible bacterial growth. MIC values > 1000 mg/mL were considered as not relevant or not active. Antibacterial drug control: ^b^ GEN: Gentamicin and ^c^ TET: Tetracycline.

## Supplementary Materials

The following are available online. NMR spectra of representative compounds in each series. Additional biological methods: tubulin staining, gamma H2A.X immunofluorescence staining and DNA intercalation assay.
