# Supplementary material for: Design and Synthesis of New 6-Nitro and 6-Amino-3,3a,4,5-Tetrahydro-2H-Benzo[g]indazole Derivatives: Antiproliferative and Antibacterial Activity
Source: Molecules. 2019 Nov 21;24(23):4236. doi: 10.3390/molecules24234236 (PMC6930490; doi:10.3390/molecules24234236)
Supplement: Supplementary file 1 [file molecules-24-04236-s001.pdf]

Supplementary Material

Design and synthesis of new 6-nitro and 6-amino-3,3a,4,5-tetrahydro-2*H*-benzo[*g*]indazole derivatives: Antiproliferative and antibacterial activity

Viviana Cuartas [1,2], María del Pilar Crespo [3], Eva-María Priego [4], Leentje Persoons [5], Dirk Daelemans [5], María-José Camarasa [4], Braulio Insuasty [1,2,*] and María-Jesús Pérez-Pérez [4,*]

1. Grupo de Investigación de Compuestos Heterocíclicos, Departamento de Química, Universidad del Valle, A. A. 25360 Cali, Colombia; viviana.cuartas@correounivalle.edu.co (V.C.)
2. Centre for Bioinformatics and Photonics-CIBioFI, Calle 13 No. 100-00, Edificio E20, No. 1069, Cali, Colombia
3. Grupo de Biotecnología e Infecciones Bacterianas, Departamento de Microbiología, Universidad del Valle, Cali, Colombia; maria.crespo.ortiz@correounivalle.edu.co (M.P.C.)
4. Instituto de Química Médica (IQM, CSIC), Juan de la Cierva 3, 28006-Madrid, Spain; empriego@iqm.csic.es (E.-M.P.); mj.camarasa@iqm.csic.es (M.-J.C.)
5. KU Leuven Department of Microbiology, Immunology and Transplantation, Laboratory of Virology and Chemotherapy, Rega Institute for Medical Research, KU Leuven, Herestraat 49, 3000 Leuven, Belgium; leentje.persoons@kuleuven.be (L.P.); dirk.daelemans@kuleuven.be (D.D.)

* Correspondence: mjperez@iqm.csic.es (M.-J.P.-P.); Tel.: +34-91-258-7516; Fax: +34-91-5644853; braulio.insuasty@correounivalle.edu.co (B.I.); Tel.: +57-315-484-6665; Fax: +57-2339-3248

Table of contents

^{1}H and ^{13}C NMR spectra of representative compounds of the different series — Pages S2-S6

Experimental procedure for tubulin binding by immunostaining, DNA damage by Gamma H2A.X immunofluorescence staining and DNA intercalation assay. — Page S7

^{1}H and ^{13}C NMR spectra of compound **7**

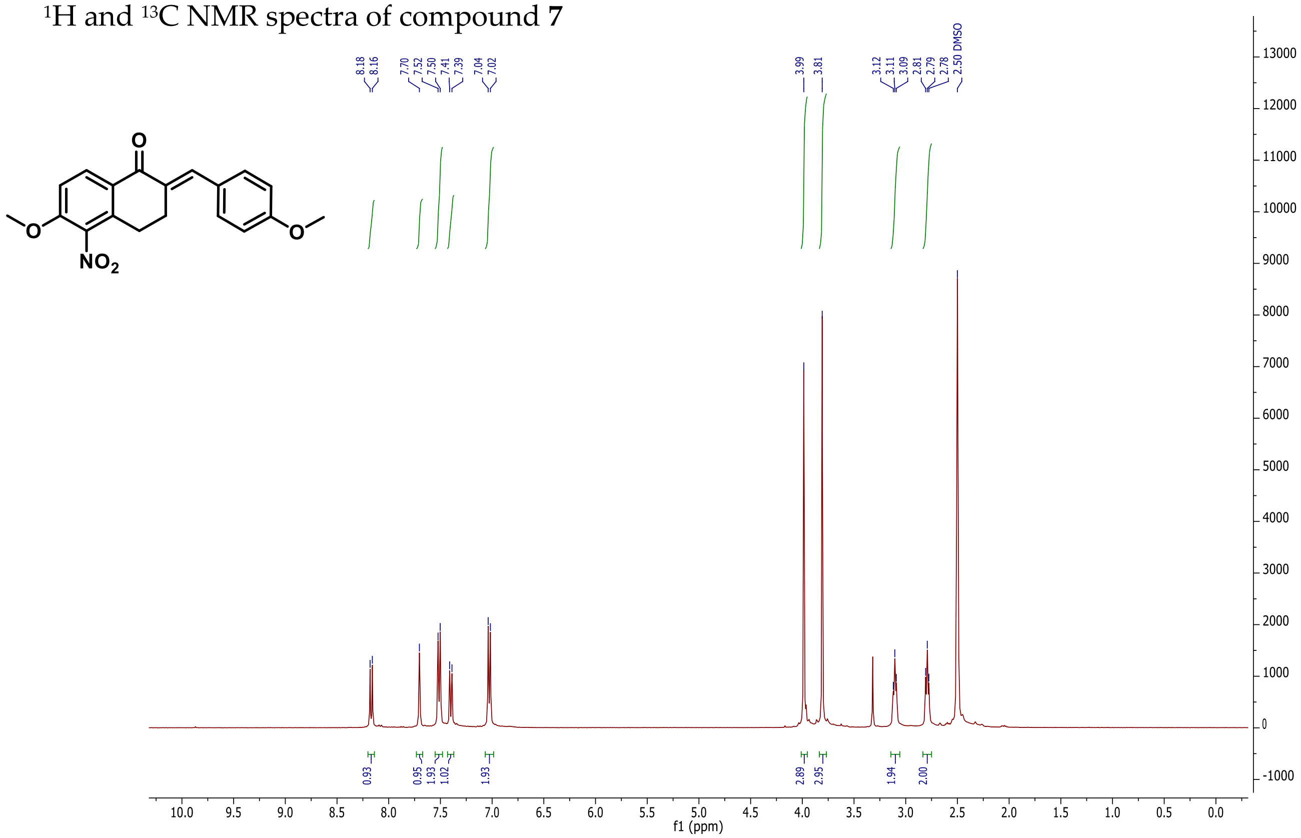

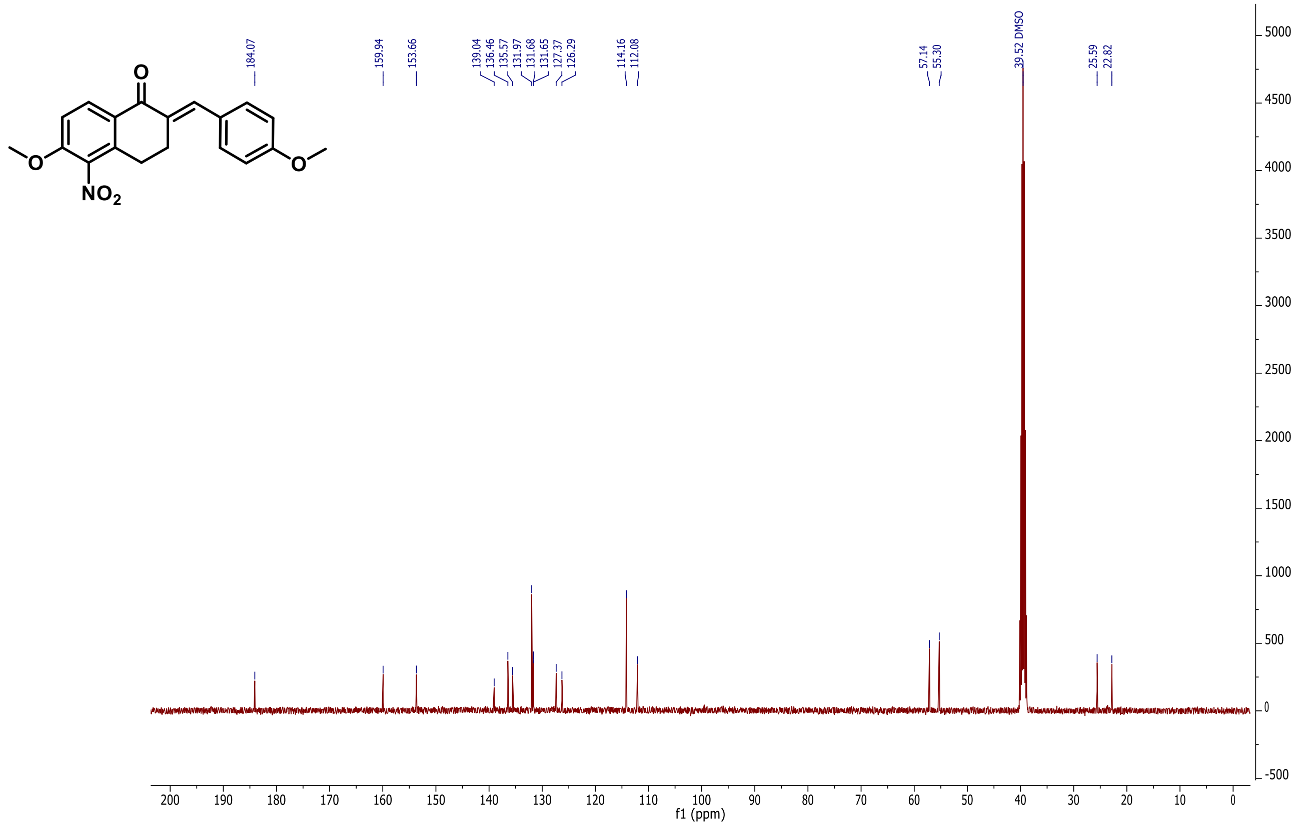

^{1}H and ^{13}C NMR spectra of compound **12a**

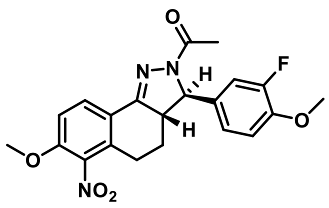

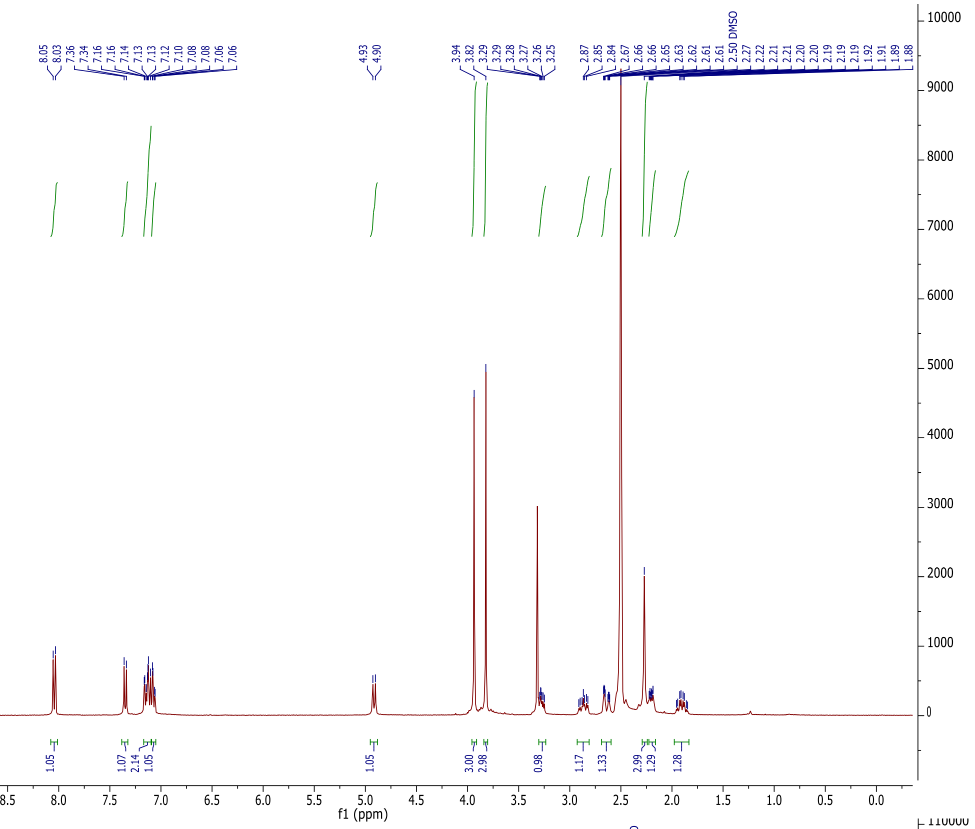

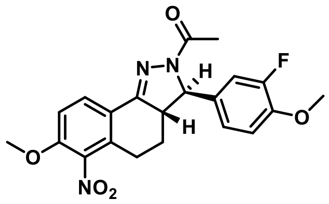

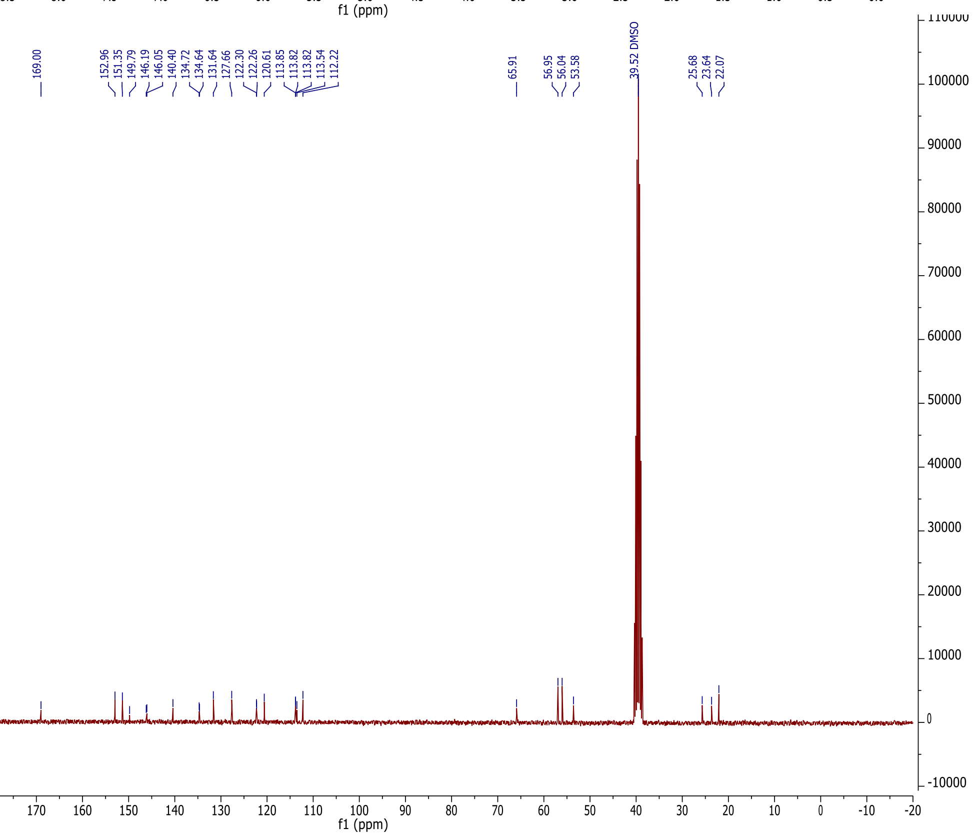

^{1}H and ^{13}C NMR spectra of compound **12b**

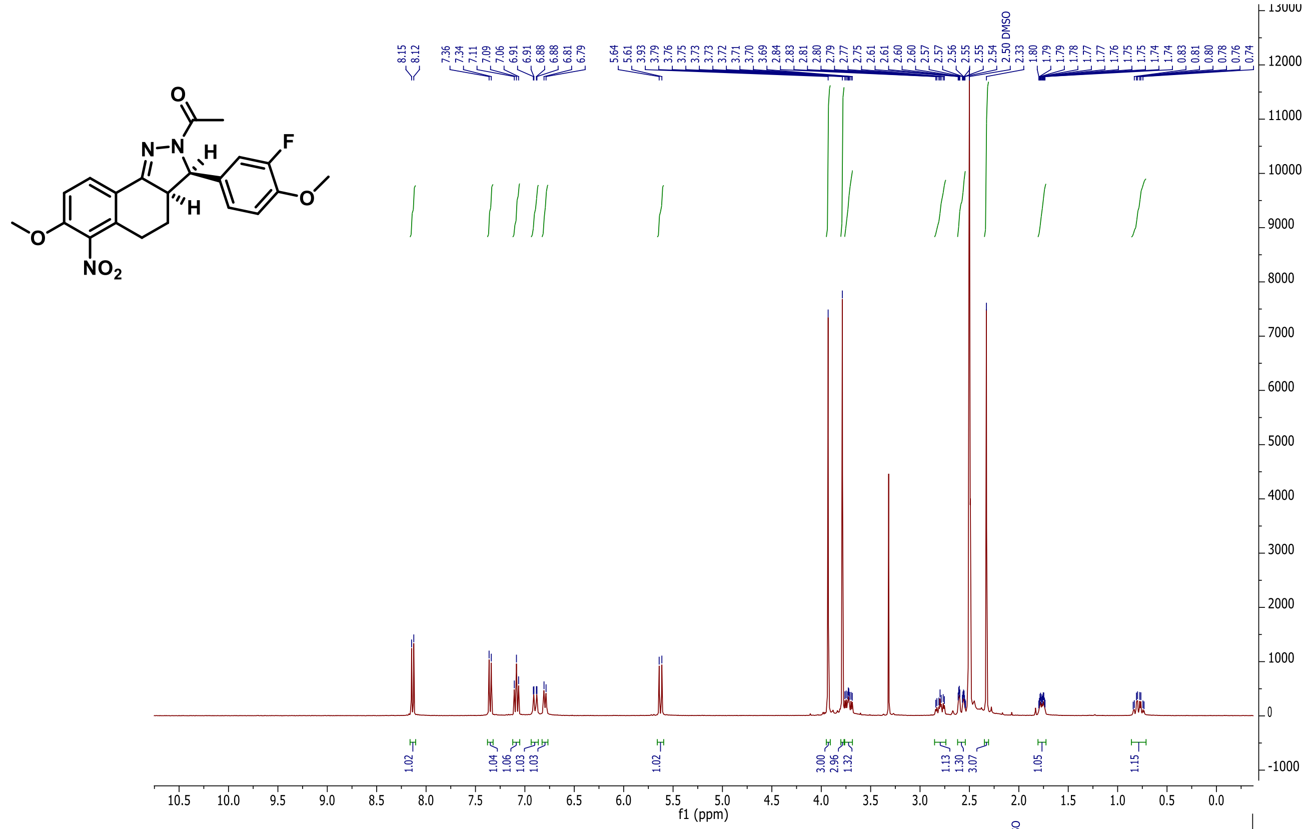

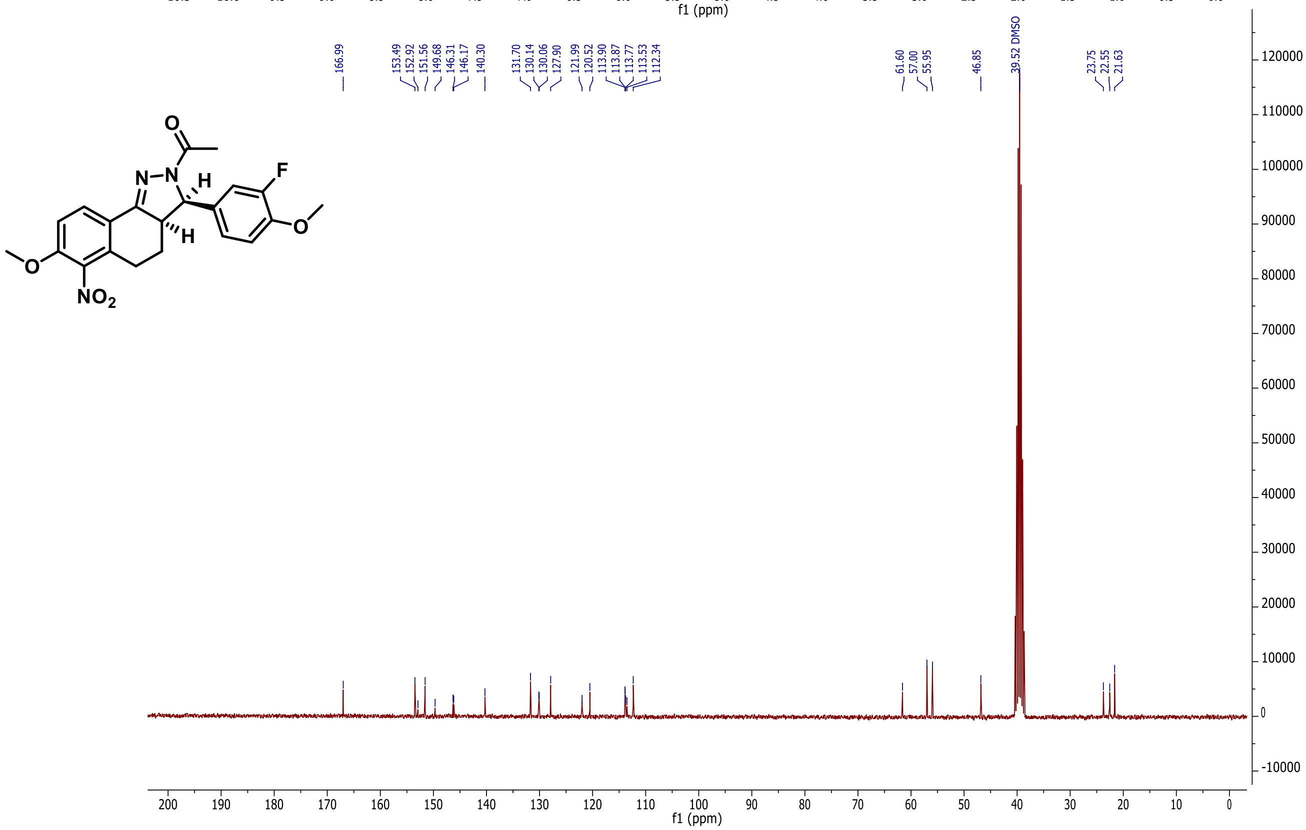

^{1}H and ^{13}C NMR spectra of compound **17a**

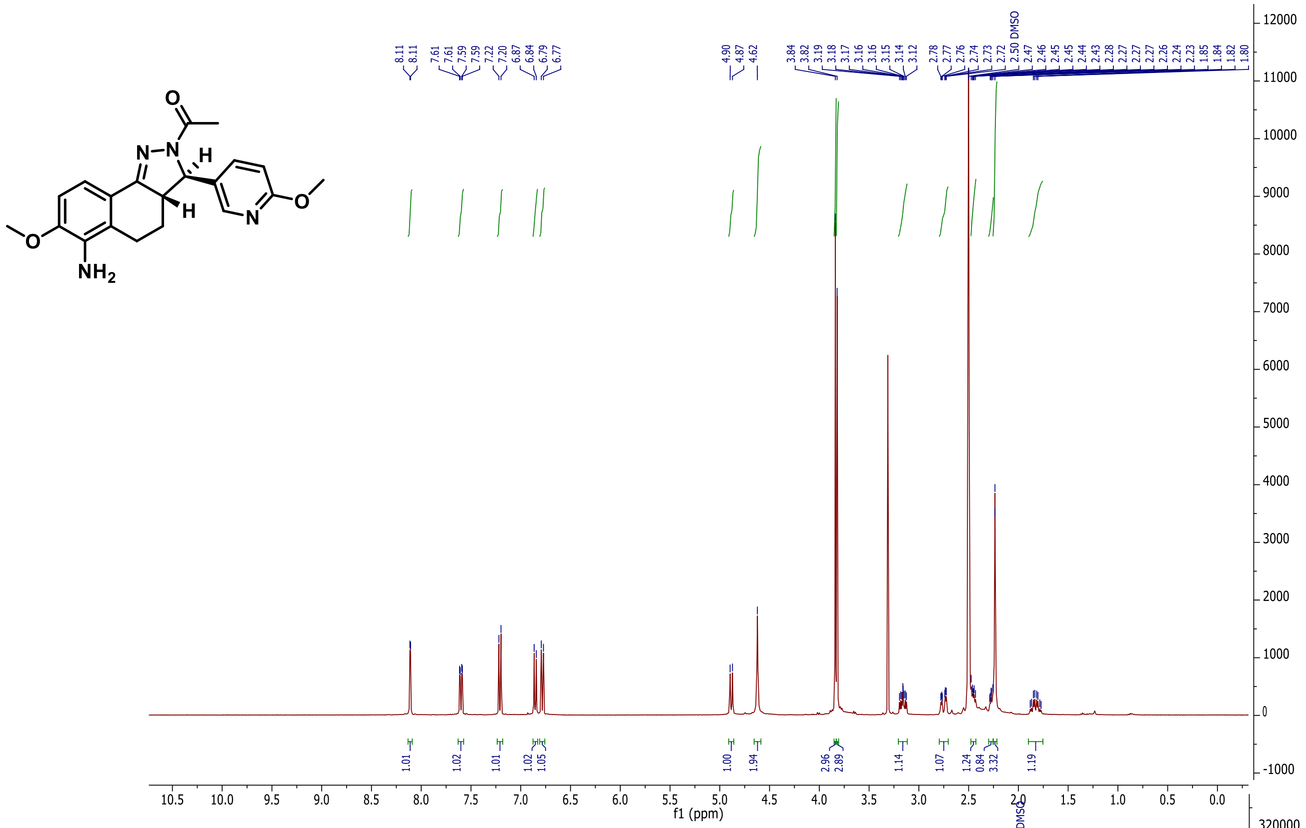

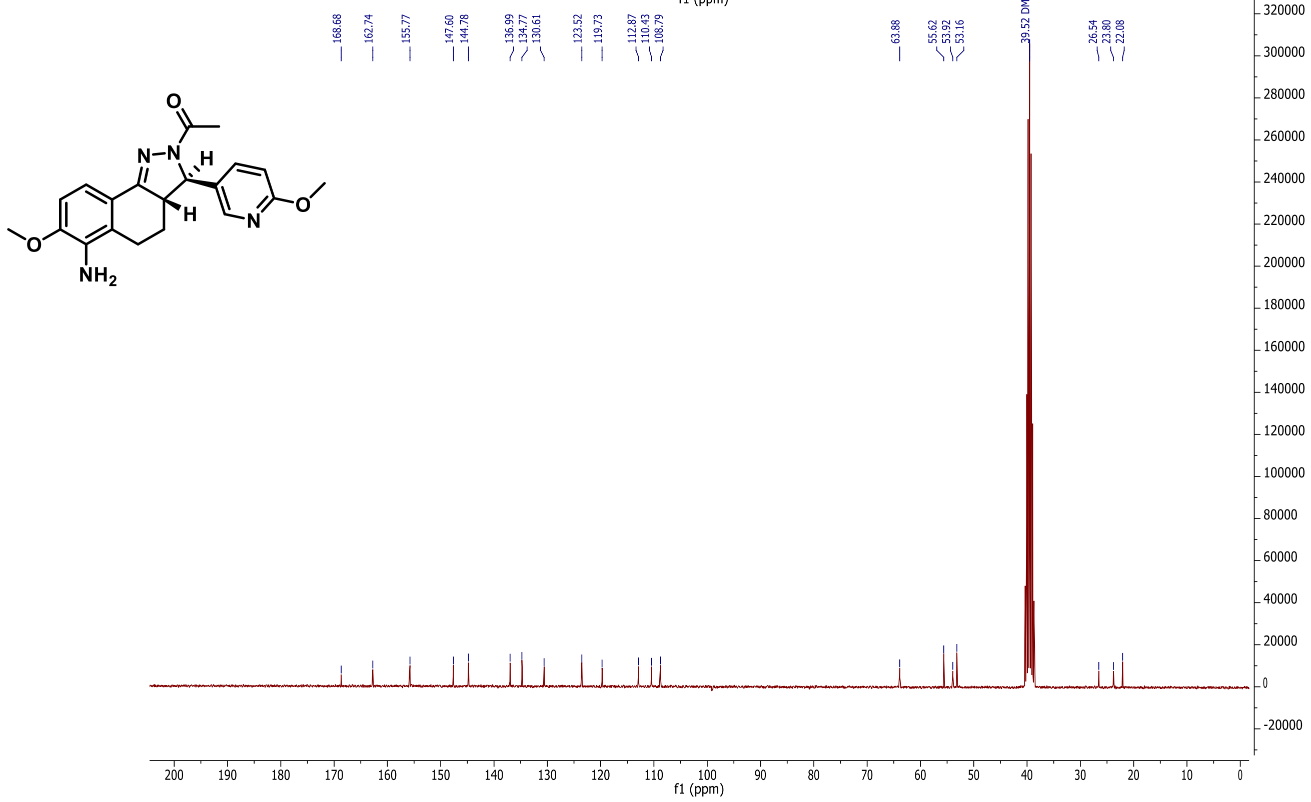

^{1}H and ^{13}C NMR spectra of compound **17b**

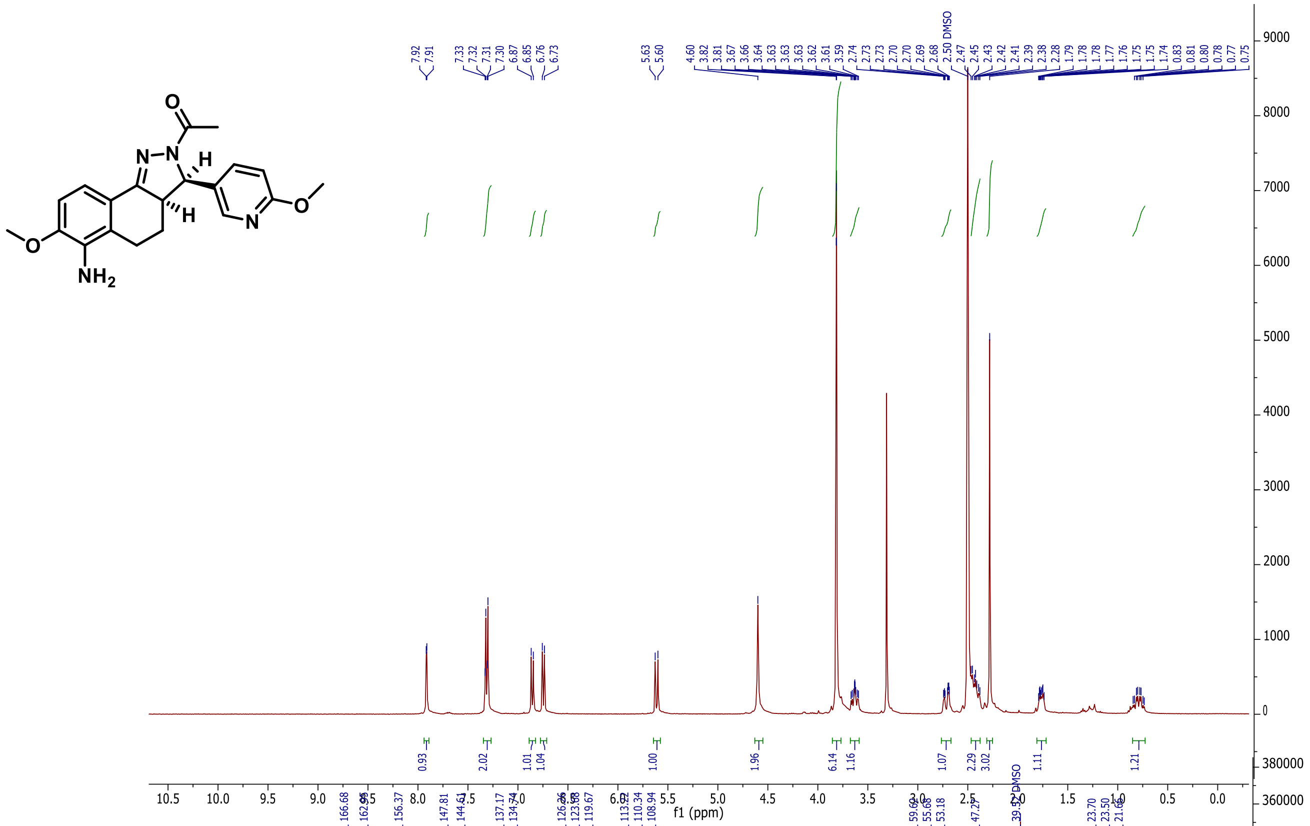

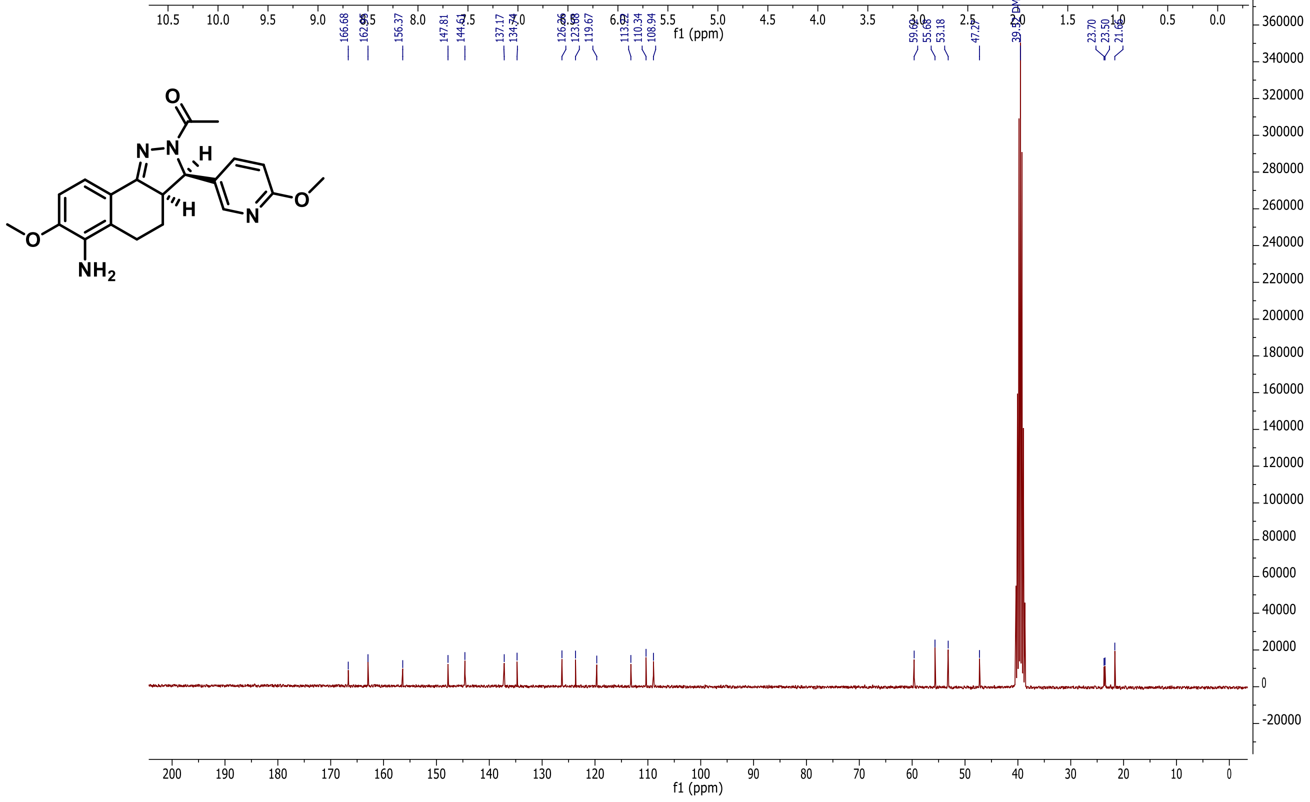

Tubulin immunofluorescence staining

Human cervix adenocarcinoma HeLa cells were seeded at 4500c/well in 96-well, black-walled, clear-bottomed tissue culture plates (Falcon). After overnight incubation they were treated with compound or carrier (DMSO) for 3h and then fixed with 4% PFA, washed and permeabilised. Further treatment was performed according to standard immunofluorescence procedures and cell nuclei were counterstained with DAPI. Employed antibodies are mouse anti-alpha tubulin and secondary goat anti-mouse IgG conjugated to Alexa Fluor® 488. Images were taken with a CellInsight CX5 high-content imager (ThermoFisher Scientific, Massachusetts, USA).

Gamma H2A.X immunofluorescence staining

Human cervix carcinoma HEp-2 cells were seeded at 4500c/well in 96-well, black-walled, clear-bottomed tissue culture plates (Falcon). After overnight incubation they were treated with compound or carrier (DMSO) for 24h and then fixed with 4% PFA, washed and permeabilised. Further treatment was performed according to standard immunofluorescence procedures, using rabbit anti-gamma H2A.X (phospho S139) and secondary goat anti-rabbit IgG conjugated to Alexa Fluor® 647. Nuclei were counterstained with DAPI, and images were taken with a CellInsight CX5 high-content imager (ThermoFisher Scientific, Massachusetts, USA).

DNA intercalation assay

The DNA intercalation assay was performed as described [1]. Acridine orange (Honeywell FLUKA) was stored at 4°C and protected from light in a 1 mM stock solution in water. 50nM acridine orange and 20µg/mL salmon sperm DNA (Invitrogen #15632-011) were incubated with compounds in HBS-EP buffer (GE Healthcare, Sweden) for 20 min. The assay was formatted for 384 well plates (Greiner #781076) in a reaction volume of 50µL per well. Mitoxantrone (MTX) was used as a positive control, and all test compounds were tested in triplo at 10 µM. Fluorescence polarization was measured using a SaFire II microplate reader (Tecan, Switzerland).

References

1. Beauchemin, C.; Moerke, N. J.; Faloon, P.; Kaye, K. M. Assay Development and High-Throughput Screening for Inhibitors of Kaposi's Sarcoma-Associated Herpesvirus N-Terminal Latency-Associated Nuclear Antigen Binding to Nucleosomes. *J Biomol Screen.* **2014**;19(6):947-58. doi: 10.1177/1087057114520973.
